# Primary Role of Functional Ischemia, Quantitative Evidence for the Two-Hit Mechanism, and Phosphodiesterase-5 Inhibitor Therapy in Mouse Muscular Dystrophy

**DOI:** 10.1371/journal.pone.0000806

**Published:** 2007-08-29

**Authors:** Akihiro Asai, Nita Sahani, Masao Kaneki, Yasuyoshi Ouchi, J.A. Jeevendra Martyn, Shingo Egusa Yasuhara

**Affiliations:** 1 Department of Anesthesiology and Critical Care, Shriners Hospital for Children, Massachusetts General Hospital, Harvard Medical School, Boston, Massachusetts, United States of America; 2 Department of Geriatric Medicine, Graduate School of Medicine, The University of Tokyo, Tokyo, Japan; University of Washington, United States of America

## Abstract

**Background:**

Duchenne Muscular Dystrophy (DMD) is characterized by increased muscle damage and an abnormal blood flow after muscle contraction: the state of functional ischemia. Until now, however, the cause-effect relationship between the pathogenesis of DMD and functional ischemia was unclear. We examined (i) whether functional ischemia is necessary to cause contraction-induced myofiber damage and (ii) whether functional ischemia alone is sufficient to induce the damage.

**Methodology/Principal Findings:**

*In vivo* microscopy was used to document assays developed to measure intramuscular red blood cell flux, to quantify the amount of vasodilatory molecules produced from myofibers, and to determine the extent of myofiber damage. Reversal of functional ischemia via pharmacological manipulation prevented contraction-induced myofiber damage in *mdx* mice, the murine equivalent of DMD. This result indicates that functional ischemia is required for, and thus an essential cause of, muscle damage in *mdx* mice. Next, to determine whether functional ischemia alone is enough to explain the disease, the extent of ischemia and the amount of myofiber damage were compared both in control and *mdx* mice. In control mice, functional ischemia alone was found insufficient to cause a similar degree of myofiber damage observed in *mdx* mice. Additional mechanisms are likely contributing to cause more severe myofiber damage in *mdx* mice, suggestive of the existence of a “two-hit” mechanism in the pathogenesis of this disease.

**Conclusions/Significance:**

Evidence was provided supporting the essential role of functional ischemia in contraction-induced myofiber damage in *mdx* mice. Furthermore, the first quantitative evidence for the “two-hit” mechanism in this disease was documented. Significantly, the vasoactive drug tadalafil, a phosphodiesterase 5 inhibitor, administered to *mdx* mice ameliorated muscle damage.

## Introduction

Duchenne Muscular Dystrophy (DMD) is caused by the lack of a gene product, dystrophin [1], and affects approximately one in 3,500 male births [2]. The skeletal muscles of DMD patients undergo slow progressive damage which leads to the onset of the disease. The precise pathophysiology is not known except for the widely accepted theory that membrane vulnerability inherent to DMD muscles plays a role [3].

Previous studies demonstrated that lack of dystrophin and its associated molecules were found to cause a defect in blood flow response in the muscle tissues [4], [5]. In response to contractile workload, normal muscles endeavor to increase the blood flow to meet the muscular metabolic demands [6]. However, when this response in blood flow is attenuated, the muscles are put under the risk of ischemia due to a lack of either sufficient supply of oxygen and nutrients or sufficient drainage of the accumulated metabolites, the pathological state of “functional ischemia” [4], [7]. Ischemia is defined as the state of blood flow decrease due to structural vascular obstruction or vasoconstriction. Functional ischemia is a status where blood flow cannot match the metabolic demand of tissues even in the absence of vascular obstruction. The balance between the demand and supply of blood flow is disturbed in both cases.

Nitric oxide (NO), a.k.a. endothelium-derived relaxing factor (EDRF), produced in skeletal muscles controls local blood flow in the muscle [8], [9] along with various other vasoregulatory molecules. In patients with DMD [4] as well as *mdx* mice (the murine equivalent) [5], [10], the sarcolemmal expression of neuronal type nitric oxide synthase (nNOS) in skeletal muscle is greatly reduced with a concomitant aberration in blood flow regulation. Various studies have reported vascular pathology [11]–[16], altered vasodilative response [17], [18], and disturbed vasodilative signaling downstream of nNOS [10]. What has not been evaluated in detail, however, is whether blood flow dysregulation due to lack of nNOS expression is a primary cause or a secondary defect of muscular dystrophies. Since nNOS knock-out mice showing similar blood flow abnormality [5] do not manifest phenotypes of muscular dystrophy [19], [20], it has been suggested that functional ischemia or lack of nNOS may be an auxiliary event but not a direct cause of the disease. Lack of a dystrophic phenotype in nNOS knock-out mice, however, means nothing more than that nNOS absence or blood flow abnormality is insufficient to cause muscular dystrophy. It is inaccurate to conclude that nNOS absence or disturbed circulation is not an essential cause of the disease. In addition to NO, tissues produce other types of vasodilatory factors, including endothelium-derived hyperpolarizing factor (EDHF) [21]. Although the identity of EDHF remains elusive, previous reports demonstrated that superoxide dismutase (SOD) produces hydrogen peroxide (H_2_O_2_), which exert EDHF-like functions [22]. Furthermore, it was previously proposed that a single factor is not enough to explain the pathogenesis of DMD and hypothesized that at least two factors are necessary to induce myofiber damage: the “two-hit hypothesis” in DMD [23].

In this study blood flow regulation in the pathogenesis of muscular dystrophy was evaluated using *in vivo* microscopic assays: we examined how blood flow responds to muscle contraction in *mdx* and control mice, whether NO/H_2_O_2_ production in muscles is attenuated in *mdx* mice, and whether augmenting the nitric oxide pathway can prevent functional ischemia and the myofiber damage in *mdx* mice. The extent of functional ischemia and the amount of myofiber damage were compared in both *mdx* and control mouse models. The phosphodiestease-5 inhibitor, tadalafil, a known vasodilator, was evaluated and found to be a therapeutic agent to reduce muscle damage.

## Results

### Control mice show a contraction-induced increase in RBC flux in muscles that is deficient in *mdx* mice

Various parameters exist for the analysis of local blood flow, including RBC flux, RBC velocity, plasma flow, blood vessel diameter and functional capillary density. Among these, RBC flux reflects local oxygen supply [24] and is a well established standard method to evaluate local blood flow. In this study, we measured the peripheral RBC flux at the intramuscular primary arterioles under *in vivo* conditions (for nomenclature of blood vessels, see supporting information, Figure S1). Observation of fluorescent RBC labeled by various dyes including PKH26GL is a well accepted method for analyzing microcirculation. To assess a possible influence of fluorescent labeling of the RBC by PKH26GL on the flow dynamics of RBCs, an alternative staining method (FITC) was used; the same pattern of kinetics (data not shown) was obtained, indicating there was no significant effect of the fluorescent staining. The basal level of the absolute crude RBC flux (stained and non-stained RBCs combined) were 8043.2±1372.5 and 10403.2±1876.0 for control and *mdx* mice (flux count per minute±S.E., N (numbers of animals) = 18 and 17, not statistically different, p = 0.31 by t-test). The validity of our labeling method is thus assured by the consistency with the previous studies reporting RBC flux at venules (12–39 µm in diameter, presumably secondary to tertiary venules) of c.a. 100,000 per minute in mice [25], and resting capillary flux of 1,800 or 1200–2000 per minute in rats [26] or in hamsters [27] (note that one primary arteriole feeds several capillaries and dozens of capillaries feed into secondary venules).

**Figure 1:**
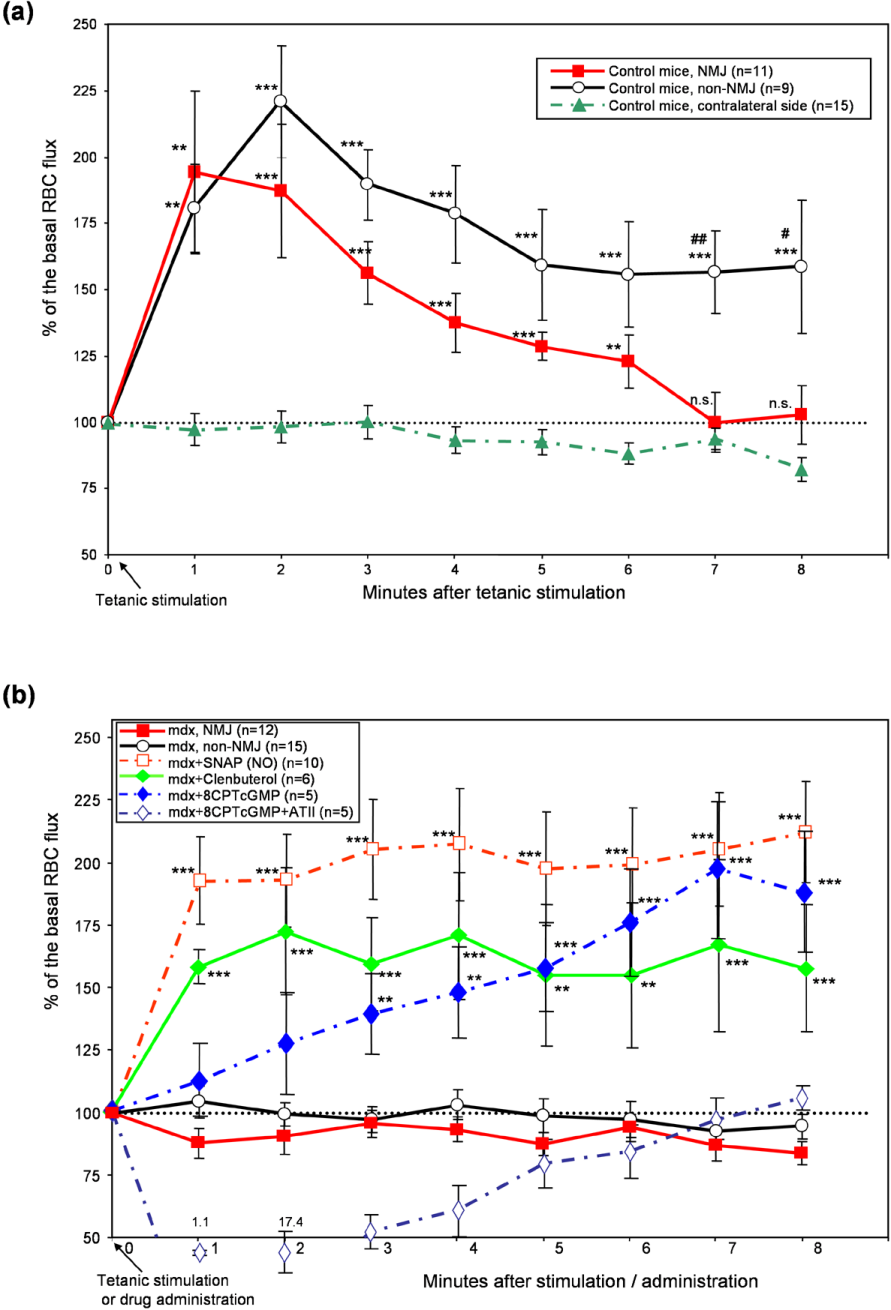
Local RBC flux is increased in the post-contraction muscle of control but not of *mdx* mice. Using *in vivo* video-microscopy, the numbers of RBC passing by through the primary arterioles (1st order) in control (a) and *mdx* (b) mice were counted and plotted against time (minutes) after a tetanic stimulation (50Hz). The y-axis represents the percent increase in the RBC number from the basal (100%) RBC flux. (a) In response to the direct tetanic stimulation on the muscles of control mice, arterioles both at junctional (NMJ, closed square, red solid line), and extrajunctional (non-NMJ, open circle, black solid line) areas showed a transient increase in the RBC. The contralateral side of the control mice (closed triangle, dash-dotted line) did not show any increase in the RBC flux, suggesting that the increase in RBC flux is the specific effect of contraction. (b) The response in RBC flux was completely absent in *mdx* mice (NMJ: closed square, red solid line, and non-NMJ: open circle, black solid line). RBC flux was increased in *mdx* mice by a local administration of SNAP (open square, red dash-dotted line), clenbuterol (closed diamond and greed solid line), or 8-CPT cGMP (closed diamond and blue dash-dotted line). Addition of 1 µg/ml of angiotensin-II (ATII) inhibited the 8-CPT cGMP-induced increase in RBC flux (open diamond, blue dash-dotted line). Addition of ATII transiently dropped the RBC flux below 50% of the basal level and the values (1.1% for 1min and 17.4% for 2 min) are indicated as numbers adjacent to each point. Unless otherwise specified, measurements were performed on arterioles in non-NMJ areas. ** and ***: Statistically significant difference from contralateral side in control mice (a) or from *mdx* mice without any treatment (b) by ANOVA (p<0.01, p<0.001, respectively). n.s. Not significantly different from contralateral side by ANOVA (a). # and ##: Statistically significant difference (p<0.05 and 0.01) between NMJ and non-NMJ by Student-t test (a). Standard errors are shown as bars at each time point. The number of individual animals in each group is indicated in the parenthesis.

As shown in Figure 1a, control mice showed an increase in RBC flux that persisted for over 8 to 10 minutes in response to contraction by 50 Hz tetanic stimulation. There were no essential differences in the pattern of RBC flux change between NMJ and non-NMJ areas in either strain of mice except for a slight difference in the time-course of RBC flux increase in control mice. Thus, all measurements were performed in non-NMJ areas hereafter unless otherwise specified. In previous experiments, high frequency stimulation at 20Hz on rat hindlimb muscles caused an increase in blood flow velocity lasting up to 14 minutes after the cessation of stimulation [28], corresponding well with our result.

Direct stimulation on one side of the sternomastoid muscle did not evoke contraction on the other side, and the RBC flux in the contralateral side of the muscle remained at the basal level (Figure 1a). This observation suggests that under the conditions utilized in this study, the stimulus on the sternomastoid muscle did not alter the cardiac output, and the increase of blood flow was specific to the local stimulation of the muscle.

The m*dx* mouse experiments showed a total absence of increase of RBC flux after tetanic stimulation both in NMJ and non-NMJ areas (Figure 1b) despite the fact that the tetanic stimuli yielded a similar extent of muscle contraction in *mdx* and control mice (Figure S2, supporting information). To examine whether the lack of response in *mdx* mice was due to the defect in blood vessels or to the defect in the signal transmission between muscles and blood vessels, we applied SNAP (S-nitroso-N-acetylpenicillamine, NO donor) locally. When SNAP was given to the resting muscles in *mdx* mice, the RBC flux was increased to a maximum of 212.4% of basal flux (Figure 1b). This increase was comparable to changes in control mice administered with the same dose of SNAP (data not shown, maximum increase up to 236.2%, N (numbers of animals) = 10, not significantly different from *mdx* mice at any time point between 0 and 8 minutes). This result suggested that vasodilatory mechanisms in the blood vessels were functional in the *mdx* mice but the signaling between skeletal muscles and blood vessels was compromised. A further confirmation of this finding was observed when a membrane permeable cGMP analogue, 8-(4-Chlorophenylthio)-guanosine 3′, 5′-cyclic monophosphate (8-CPT cGMP) was locally applied. This drug caused a slow increase in the RBC flux in *mdx* mice, reaching a similar extent of response to that seen in the SNAP group by 7 minutes after administration. Given that the vasodilatory effect of NO is through a cGMP-dependent pathway [29]–[31], the difference in the kinetics of RBC flux increase between groups with SNAP and with 8-CPT cGMP is likely due to the difference in the speed of the drugs to reach their target cells. The effect on RBC flux increase by 8-CPT cGMP is not a non-specific irreversible vaso-action, but is likely a specific physiological regulation, because this response was completely antagonized by a further addition of physiological concentration of a vasoconstrictor, angiotensin-II (ATII). Because β2-adrenergic agonists have a different mechanism of vasodilation that does not involve NO [32], we examined whether clenbuterol causes vasodilation in *mdx* mice. When clenbuterol was locally administered instead of SNAP, *mdx* mice showed an increase in RBC flux, albeit to a lesser extent (maximum up to 172%, Figure 1b). The result from the clenbuterol experiment confirms that the mechanism of vasodilation inherent to the vascular smooth muscle is functional in *mdx* mice.

### Nitric Oxide and hydrogen peroxide production in response to muscle contraction is attenuated in *mdx* mice

**Figure 2:**
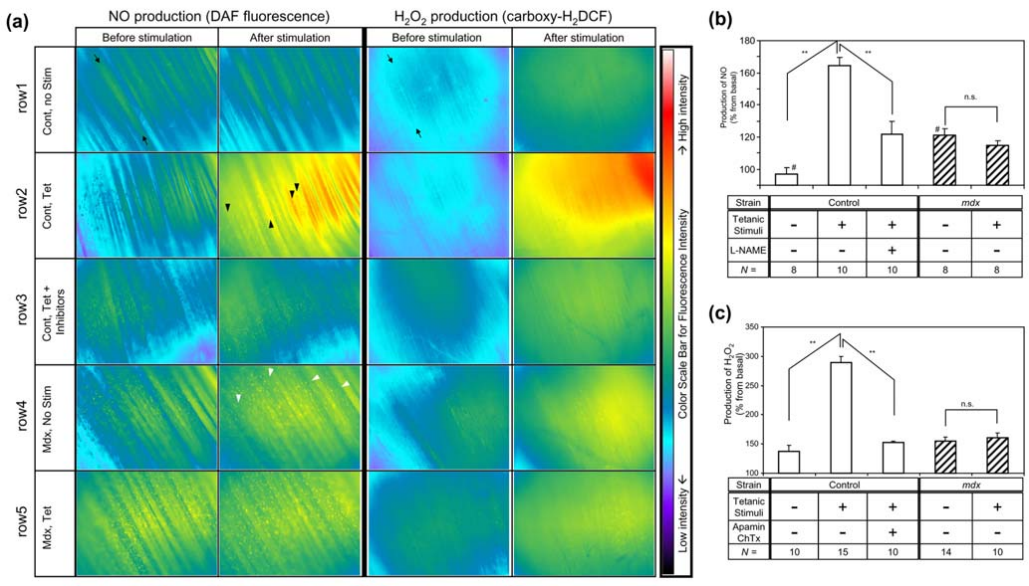
* In vivo* microscopic measurement of muscle production of NO and H_2_O_2_ in control and *mdx* mice after tetanic stimulation. (a) *In vivo* microscopic views of fluorescent signal (100×) are presented with pseudocolors added according to the fluorescent intensity of the signals (a). Warmer colors correspond to higher intensity (note scale bar on right side). NO or H_2_O_2_ produced by stimulated myofibers reacts with DAF-FM (left column) or H_2_-DCFDA (right column) respectively, and releases a fluorescence signal. The longitudinal area between the two black arrows in the microscopic image corresponds to an individual myofiber (row1, Cont, no Stim). Myofibers in the control muscles produce a prominent amount of NO (left) and H_2_O_2_ (right) in response to tetanic stimuli (50Hz), shown as Cont, Tet (row 2). The spot-like staining showed an increased production of NO in response to muscle contraction (“Cont, Tet” in the left column, examples pointed by a black arrow head), but were not prominent with H_2_O_2_ signal (right column). A non-specific NOS inhibitor L-NAME (row3, left), or combination of EDHF inhibitors apamin and charybdotoxin (row 3, right), perturbed production of NO or H_2_O_2_, respectively, after tetanic stimulation (Cont, Tet+Inhibitors). Although the basal level of NO production in the *mdx* muscle is high (row 4, left column, *Mdx*, no Stim, p = 0.004 by Student-t test), muscles in these mice do not show an increase in the NO (row 5, left) or H_2_O_2_ (row 5, right) production in response to muscle contraction (*Mdx*, Tet). *Mdx* mice showed greater numbers of spot-like staining for NO (examples pointed by a white arrow head) as compared to control mice, but these spots did not show an increase in intensity after muscle contraction. (b&c) The quantification data of the detected signal of NO (b) and H_2_O_2 _(c) in the sternomastoid muscles are shown. Average fluorescence released by myocytes was calculated by densitometry of the captured images from *in vivo* microscopy on different mice (the numbers of animals in each group indicated in the bottom row). The y-axis represents the percent increase in the arbitrary fluorescence unit per 30 (b) or 60 (c) minutes of observation. **: Statistically significant by ANOVA (P<0.01). n.s.: Not statistically significant. #: Statistically significant between the basal level of control and *mdx* mice (P<0.05). Error bars show standard error of each value.

*In vivo* production of vasodilatory molecules (i.e. NO and H_2_O_2_) was measured in the sternomastoid muscles in *mdx* and control mice to investigate the mechanisms for functional ischemia in *mdx* mice. Direct tetanic stimulation on the sternomastoid muscle induced a marked elevation in the level of NO in the myofibers (Figure 2 a&b). When L-NAME (*N*ω-Nitro-L-arginine methyl ester), a non-specific NOS inhibitor, was applied, production of NO from muscle fibers was reduced by approximately 67.6% of the net increase. This result demonstrated that the majority of the detected fluorescent signal was specific to the produced NO. The fact that exogenously applied L-NAME cannot suppress all the intracellular NO production is consistent with previous studies [33]. The effects of muscle contraction on NO production between control and *mdx* mice were compared (Figure 2 a&b). The basal level of NO in the resting muscle of *mdx* mice was higher than control mice, but the increase in the NO production after muscle contraction was completely abolished in *mdx* mice. Production of NO from non-myofiber cells was noted (black and white arrow heads in Figure 2a).

Although the identity of EDHF has not been fully confirmed, previous studies demonstrated the production of hydrogen peroxide (H_2_O_2_), detected by the fluorescence of carboxy-H_2_-DCFDA (2′,7′- dichloro-dihydrofluorescein diacetate), has the EDHF-like function [22]. In this study, H_2_O_2_ production in the sternomastoid muscle was compared between control and *mdx* mice by using carboxy-H_2_-DCFDA. Direct tetanic stimulation on the muscle induced the production of H_2_O_2_ inside the myocytes in the control mice (Figures 2a&c). It is well established that pharmacological agents, including the combination of apamin and charybdotoxin [34], inhibit EDHF by antagonizing calcium activated potassium channels on vascular endothelial cells in *ex vivo* experiments [34], [35]. The production of H_2_O_2_ by myofibers was inhibited by the combined application of apamin and charybdotoxin to the muscle superfusative solution (Figures 2a right 3rd panel, 2c 3rd column). The increase in the amount of H_2_O_2_ detected after tetanic stimulation was attenuated in the *mdx* mice (Figures 2a right bottom panel, 2c far right column). These experiments demonstrate that in the sternomastoid muscle of control mice, both NO and H_2_O_2_ are increased after tetanic stimuli, while in *mdx* mice tetanic stimulation fails to increase the production of both molecules, a potential cause of the disturbed regulation of RBC flux.

### Pharmacological reversal of functional ischemia prevents exercise-induced myofiber damage in *mdx* mice

**Figure 3:**
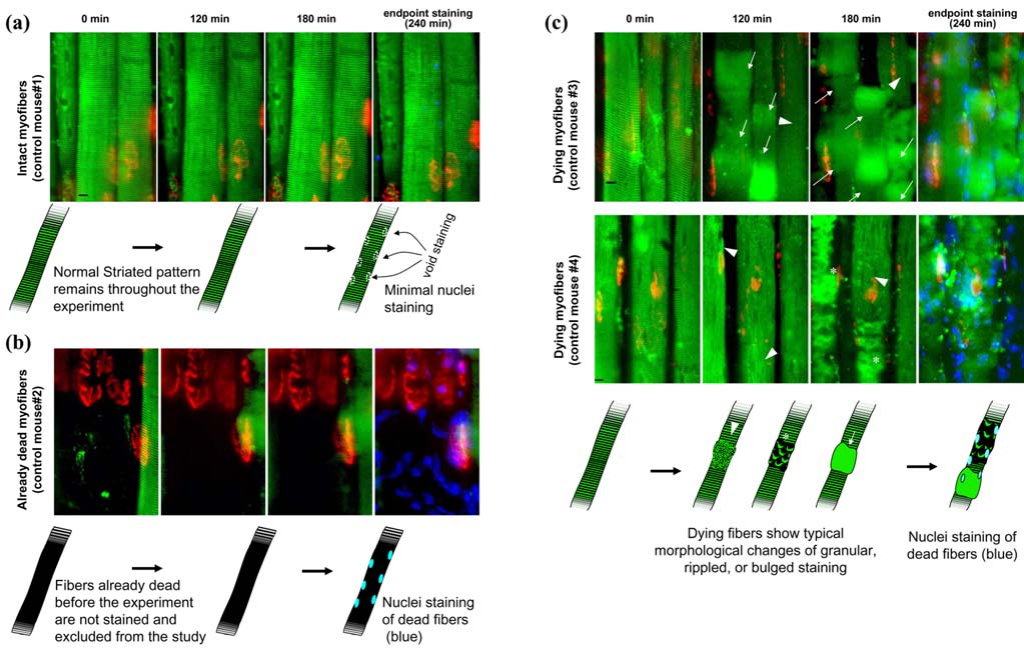
* In vivo* microscopic assay of myofiber damage. Identical positions of the myofibers were traced by examining the anatomy of the individual myofibers (stained green), and/or the shape and relative location of NMJs (stained red by BTX-Alexa Fluor 594). (a) Intact cells in control mice with no treatment showed a normal striated pattern of staining of M/ER with DiOC_6_ (Intact myofibers, control mouse#1). (b) Already dead myofibers (killed by electrical ablasion 1 hour before the experiment) are not stained and excluded from the study. (c) When cells are induced to death by combination of severe ischemia (L-NAME, apamin, charybdotoxin, and vascular oppression) and strenuous contraction (12 times repeat of tetanic stimuli), distribution of these DiOC_6_-stained compartments becomes granular (arrow heads) or bulged (arrows), or exhibit a rippled pattern (asterisk). Each image is in focus. Cell death identified by abnormal DiOC_6_ labeling was confirmed by dye-exclusion staining with short exposure to Hoechst33258 (blue color at endpoint in each group). The black scale bar in the figure represents 10 µm.

Experiments were designed to determine whether improvement of microcirculation by replenishing NO in *mdx* mice (Figure 1b) can prevent muscle contraction-induced cell death. Using DiOC_6_, a membrane potential-dependent dye that is incorporated into mitochondria and endoplasmic reticulum (M/ER) only in live cells (Figure 3a), we followed the chronological change in the morphology of myofibers *in vivo* and counted the number of damaged myofibers. An advantage of this technique is that fibers already dead before treatments are not stained (Figure 3b), while fibers damaged after the staining can be detected (Figure 3c). Thus, it became possible to evaluate the specific effect of muscle contraction and/or pharmacological intervention.

Intact muscle fibers showed a green fluorescent signal of clusters of M/ER in a striated pattern (Figure 3a). For quantification of myofiber damage, we counted numbers of damaged loci (locus) instead of numbers of the damaged fibers, because there are different types of damage observed along the length of a myofiber (see Methods for detail). Dying muscle fiber loci both in control (Figure 3c) and *mdx* (not shown) mice revealed a disturbed intracellular distribution pattern of the green fluorescence. Cell death was confirmed by dye-exclusion staining (endpoint blue color in Figure 3) and annexin-V staining (Figure S3, supporting information). This result from our *in vivo* study confirms earlier morphological analyses of mitochondrial abnormality in skeletal muscle cell death observed in toxin-induced [36], [37] and muscular dystrophy [38] mouse models. The myofiber loci with signs of damage (granular, ripple, and bulged staining by DiOC_6_) never returned to the normal striated staining after a prolonged time-lapse observation up to 12 hours. The morphological changes stated above are considered irreversible and myofiber loci manifesting these characteristics are indeed dying (for criteria of fiber loci counting, see supporting information, Figure S4a&b). The staining procedure did not affect muscle viability and intact myofibers retained their normal striated pattern of M/ER during the time of observation (Figure 3a).

**Figure 4:**
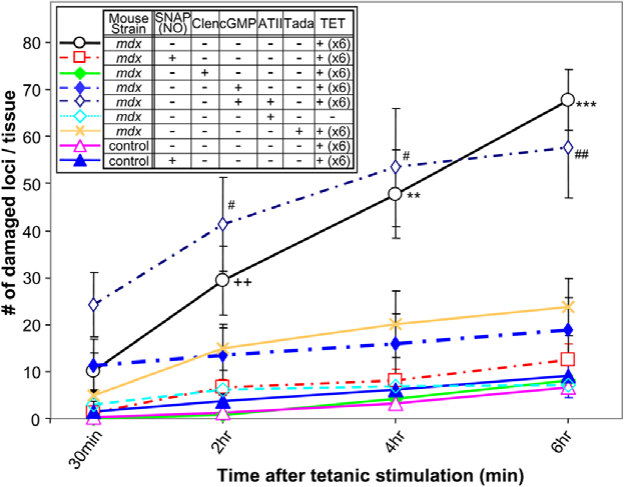
Replenishing NO prevents contraction-induced myofiber cell death. *Mdx* mice (open circle and black solid line, N = 6) showed progressive increase of damaged loci over the time course of 6 hours after a 6 times repeat of the tetanic stimuli (X-axis: time after tetanic contraction. Y-axis: damaged myofiber loci counted throughout the entire tissue). For quantification of myofiber damage, the numbers of damaged sites (loci) were counted instead of numbers of the damaged fibers, because there are various types and different locus onset of damage along the length of a myofiber (see Methods for detail). Administration of SNAP (NO donor, 100 µM) prevented the *mdx* mice fibers from undergoing contraction-induced damage (open square and red dash-dotted line, N = 5). In the groups where 8-CPT-cGMP (“cGMP”, 500 µM, closed diamond and blue dash-dotted line, N = 5), clenbuterol (“Clen”, 0.05 mg/ml, closed diamond and green solid line, N = 4), or tadalafil (“Tada”, 4 mg/kgBW, cross and orange solid line, N = 5) was given during muscle contraction, the increase in myofiber damage was abolished. Addition of 1 µg/ml of angiotensin-II (ATII) inhibited the myofiber protective effect by 8-CPT-cGMP (open diamond and blue dash-dotted line, N = 6). ATII alone did not cause myofiber damage (open diamond and blue dotted line, N = 4). Control mice with (closed triangle and blue solid line, N = 4) or without (open triangle and pink solid line, N = 6) SNAP administration did not show increase in myofiber damage. Statistical differences are indicated between *mdx* without drug treatment (black open circle) and all other groups except for 8-CPT cGMP plus ATII (**: p<0.01, ***:p<0.001), or all other groups except for 8-CPT cGMP or 8-CPT cGMP plus ATII (++: p<0.01) by ANOVA. There was a statistically significant difference between groups with 8-CPT cGMP and with 8-CPT cGMP plus ATII (#: p<0.05, ##: p<0.01) by Student-t test. There was no significant difference between groups without any treatment (black open circle) and with 8-CPT cGMP plus ATII (blue open diamond) at any time point. Error bars in the graph are the standard errors to each value. All the drugs are removed and tissues are washed after muscle contraction. N refers to the number of animals used for each treatment.

Tetanic stimulation (6 times repeat, 5 seconds duration at 50 Hz) induced progressive myofiber damage in the *mdx* mice (Figure 4, open circle and black line, “*mdx*”), consistent with previous reports of mechanical weakness of the myofibers from muscular dystrophy subjects [39]. At 6 hours after tetanic stimulation, 67.67±6.57 (count±S.E.) myofiber loci were damaged out of the entire field of observation. The total numbers of loci was 350 to 400 (70 to 80 myofibers scanned 5 times).

We evaluated whether reversal of functional ischemia can attenuate this contraction-induced myofiber damage. As demonstrated in Figure 1b, an NO donor, SNAP, can improve muscle blood flow in *mdx* mice. When SNAP was locally applied to *mdx* mice during muscle contraction cell death was completely abolished (Figure 4, open square and red dash-dotted line, “*mdx*+NO”). Microscopic images of these experiments are provided in Figure S5a (supporting information).

These observations from the SNAP experiments support the hypothesis that functional ischemia is necessary and plays a primary role in contraction-induced myofiber damage in *mdx* mice and can be prevented by augmentation of NO. To further investigate whether this cytoprotective effect involves a cGMP-dependent pathway, the effect of 8-CPT cGMP was examined in the same cell death experiment since this drug increases RBC flux in *mdx* mice (Figure 1b). The contraction-induced myofiber damage was successfully prevented (Figure 4, closed diamond and blue dash-dot line). The myofiber protective effect by 8-CPT cGMP was likely mediated by its vasoregulatory potential, because this beneficial response was completely inhibited when ATII was further added at the concentration that antagonized the increase in RBC flux (Figure 4, open diamond with blue dash-dot line). The inhibitory effect by ATII on cGMP-mediated cytoprotection is likely through the vascular regulation, but not by its direct catabolic function on myofibers, because ATII alone did not have a cytotoxic effect on myofibers (Figure 4, open diamond with light-blue dash line). When the β2-adrenergic agonist clenbuterol was locally administered instead, before and during tetanic stimulation, contraction-induced myofiber damage was attenuated (Figure 4, closed diamond and green solid line) although β2-adrenergic agonists are known to have different vasodilatory mechanisms from NO/EDHF [32]. Since previous studies reported elevated PDE5 activity in the skeletal muscle samples from *mdx* mice [40] and decreased cGMP production [10], we hypothesized that administration of PDE5 inhibitor will increase the amount of cGMP, and therefore prevent myofiber damage as predicted from the data with 8-CPTcGMP. Tadalafil (4 mg/kgBW ) was applied to the *mdx* mice via a gastric tube 60 minutes before the start of tetanic stimulation. Administration of tadalafil lowered the amount of myofiber damage (Figure 4, cross mark and orange solid line). Placebo treatments did not prevent the myofiber damage (data not shown).

### Is functional ischemia sufficient to cause contraction-induced myofiber damage?

**Figure 5:**
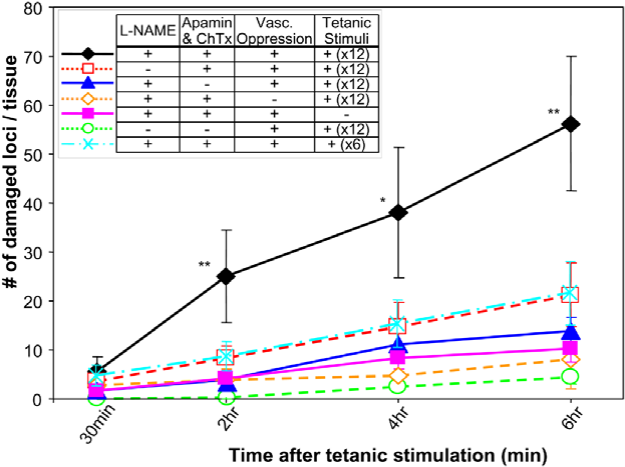
Severe ischemia induced by pharmacological interventions results in myofiber damage in control mouse receiving repeated tetanic stimulation. Numbers of damaged myofiber loci (y-axis) were counted throughout the entire tissue using *in vivo* microscopy and plotted against time after tetanic contraction (x-axis). For quantification of myofiber damage, the numbers of damaged sites (loci) were counted instead of numbers of the damaged fibers, because there are different types of damage observed along the length of a myofiber (see Methods for detail). When ischemia stress was provided by applying L-NAME, apamin plus charybdotoxin (ChTx), and vascular oppression, control mice showed progressive increase in the amount of muscle damage (closed diamond and black solid line) under the application of increased stimuli (12 times). However, when L-NAME (open square and red dotted line), apamin plus charybdotoxin (closed triangle and blue solid line), or vascular oppression (open diamond and orange dotted line) was lacking, a comparable amount of myofiber injury was not achieved. Even when all reagents were present, the amount of myofiber damage was minimal without tetanic contraction (closed square and pink solid line). Tetanic stimulation and vascular oppression alone did not cause significant myofiber destruction (open circle and green dotted line). Six times stimuli applied in control mice (X mark and blue dash-dotted line) did not induce an equivalent amount of myofiber damage as compared to 12 times control (black diamond) or to 6 times *mdx* mice (Figure 4, open black circle). N = 5 mice for each treatment. *:p<0.05, **:p<0.01, by ANOVA. Standard errors are shown as bars added to each point.

Experiments were designed to evaluate whether skeletal muscle cell death similar to those observed in *mdx* mice can be simulated by inflicting artificial functional ischemia on the wild-type animals. Only when L-NAME (inhibition of NO production), apamin plus charybdotoxin (inhibition of EDHF), and vascular oppression was given concomitantly, a twelve-times repeat of tetanic stimuli produced 56.2±13.69 (count±S.E.) damaged myofiber loci at 6 hours post tetanic stimulation (closed diamond and black line in the Figure 5). Comparable myofiber damage was not observed in the absence of any one of the following reagents/interventions: L-NAME (Figure 5, open square and red dotted line), apamin plus charybdotoxin (closed triangle and blue line), vascular oppression (open diamond and orange dotted line), or tetanic stimulation (closed square and pink line). Tetanic stimulation and vascular oppression alone did not cause prominent myofiber damage (open circle and green dotted line). A six time repeat of tetanic stimuli caused less amount of myofiber damage (21.60±6.52 (count±S.E.) loci at 6 hours, cross and cyan dash-dotted line). Detailed microscopic images of each group are provided in the Figure S5b (supporting information). Our pharmacological intervention did not revert the abnormal fragmented morphology of NMJs in *mdx* mice (Figure S5a, red staining) to normal pretzel-like shape (Figure S5b), or vice versa in control mice, during the 6 hours of observation.

**Figure 6:**
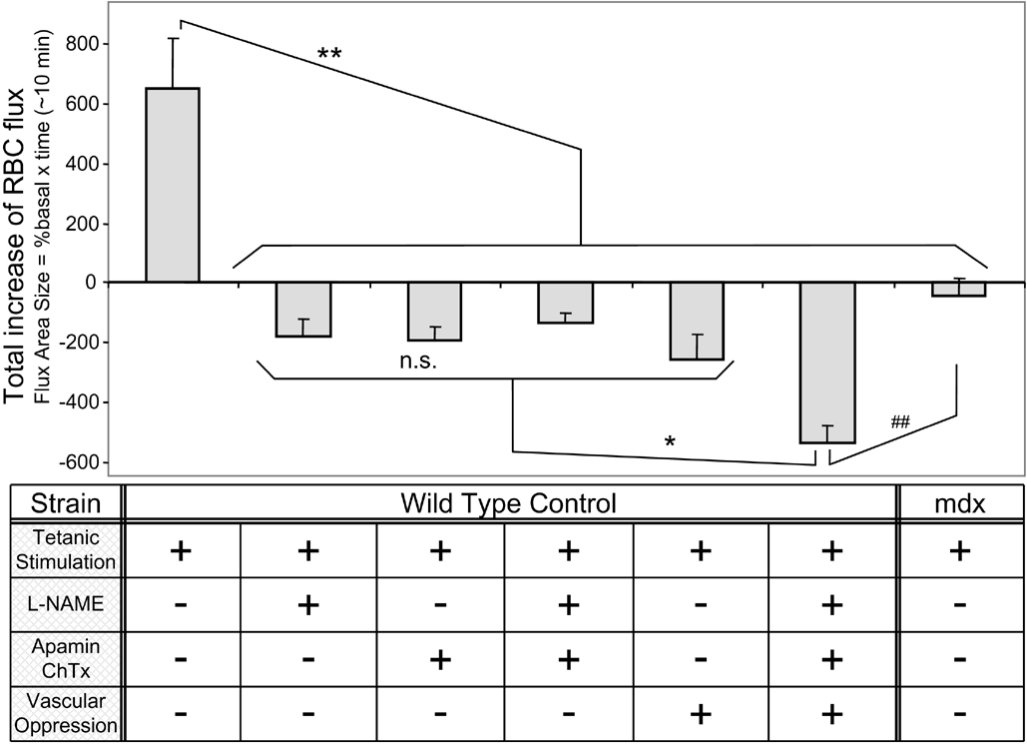
Comparison of total RBC flux increase in control and *mdx* mice in response to tetanic stimulation and pharmacological agents. Total RBC flux increase was calculated as an integral of % change in RBC flux after stimulation. Tetanic stimulation increased the flux to 653.2 (%×min, far left column) in control mice. Administration of L-NAME (2nd column), apamin plus charybdotoxin (3rd), or vascular oppression (5th) suppressed the increase in RBC flux. When all the above treatments were combined, RBC flux was further suppressed (6th). The level of ischemia in the control mice receiving all the combination (6th) was more severe as compared to *mdx* mice (far right). N = 8 mice, for each treatment. * and **: Statistically significant difference from all other treatments (p<0.05 and 0.01, ANOVA). ##: Statistically significant difference between the two groups (p<0.01, t-test). Standard error bars are added to the histogram.

The amount of functional ischemia achieved by various treatments were quantified by integrating the total change of RBC flux during the 10 minutes after tetanic stimulation (calculations shown in supporting information, Figure S6) to evaluate whether functional ischemia alone was enough to explain the contraction-induced myofiber damage in *mdx* mice. Functional ischemia is defined as a pathological state when there is a lack of normal response in blood flow in post-contraction muscles: in our experiment, *mdx* mice did not show an increase of RBC flux in response to tetanic stimulation (Figure 6, far right column). Treatment of control mice with L-NAME, apamin plus charybdotoxin, or vascular oppression individually caused functional ischemia to a similar extent observed in *mdx* mice, but caused a larger decrease in RBC flux when all were combined (Figure 6). In the case where the combination of treatments caused a significant drop in RBC flux, such a pathological state fits in the definition of “severe ischemia”, and not functional ischemia. Theoretically, a complete obstruction of the arteriole under observation will result in the value of negative 950. In our experiments, the severest ischemia we observed had a value of negative 537 (Figure 6, all drug combination, 2nd column from right) and still maintained a certain level of blood flow. These experiments suggest that functional ischemia alone is not enough to cause myofiber damage.

### Vascular therapy with tadalafil, a phosphodiesterase-5 inhibitor

**Figure 7:**
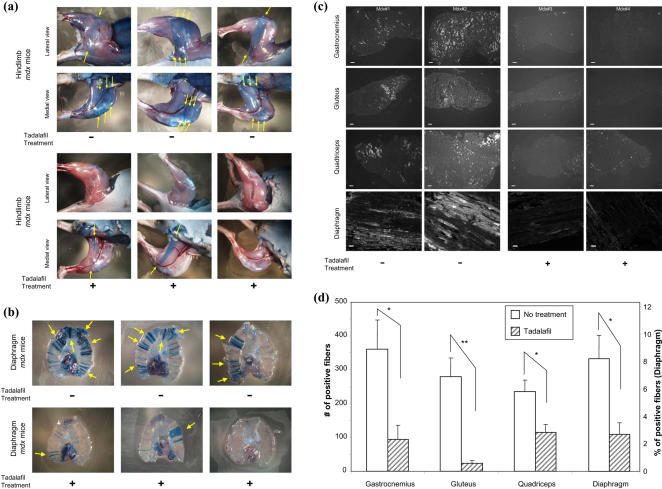
Evans Blue staining of the damaged fibers in the hindlimb and the diaphragm muscles of *mdx* mice (4 weeks old). (a&b) Six hours after the injection of Evans Blue dye, the extent of myofiber damage as indicated by blue staining was observed in the superficial hindlimb muscles (a) and the diaphragm (b). Three mice from each group are shown (a and b). Compare lateral and medial views of the stained fibers (arrows) in mice without (−) and with (+) tadalafil treatment (a). Mice without tadalafil treatment (−) show extensive blue staining (arrows in a and b). Tadalafil treatment (+) ameliorated the damage in the same muscle tissues, although it did not completely suppress the myofiber damage in some *mdx* mice. (c) Gastrocnemius, gluteus maximus, quadriceps, and diaphragm muscles were harvested and cryosectioned for fluorescence microscopic observation. In the non-treated group (Mdx#1 and #2 in c), all the muscles studied showed increased numbers of damaged myofibers stained by the injected dye (high fluorescence signals are shown in white). Tadalafil treatment reduced the numbers of damaged myofibers (Mdx#3 and #4 in c). The white scale bar at the bottom of images represents 100 µm for gastrocnemius, gluteus, and quadriceps, and 50 µm for diaphragm. (d) The number of positively stained damaged myofibers (in c) were counted and shown as bar graphs for the entire gastrocnemius, gluteus, and quadriceps muscles. For diaphragm muscles, the positively stained myofibers are shown as percentage of the total fiber count. *Mdx* mice without treatment (N = 10, white columns) showed extensive amount of myofiber damage. Tadalafil treatment (N = 8, columns shaded with hatched lines) showed a statistically significant decrease in the amount of damaged myofibers in gastrocnemius, gluteus maximus, and quadriceps muscles. *: p<0.05, **<p<0.01 by t-test. N refers to the number of animals used for each treatment.

**Figure 8:**
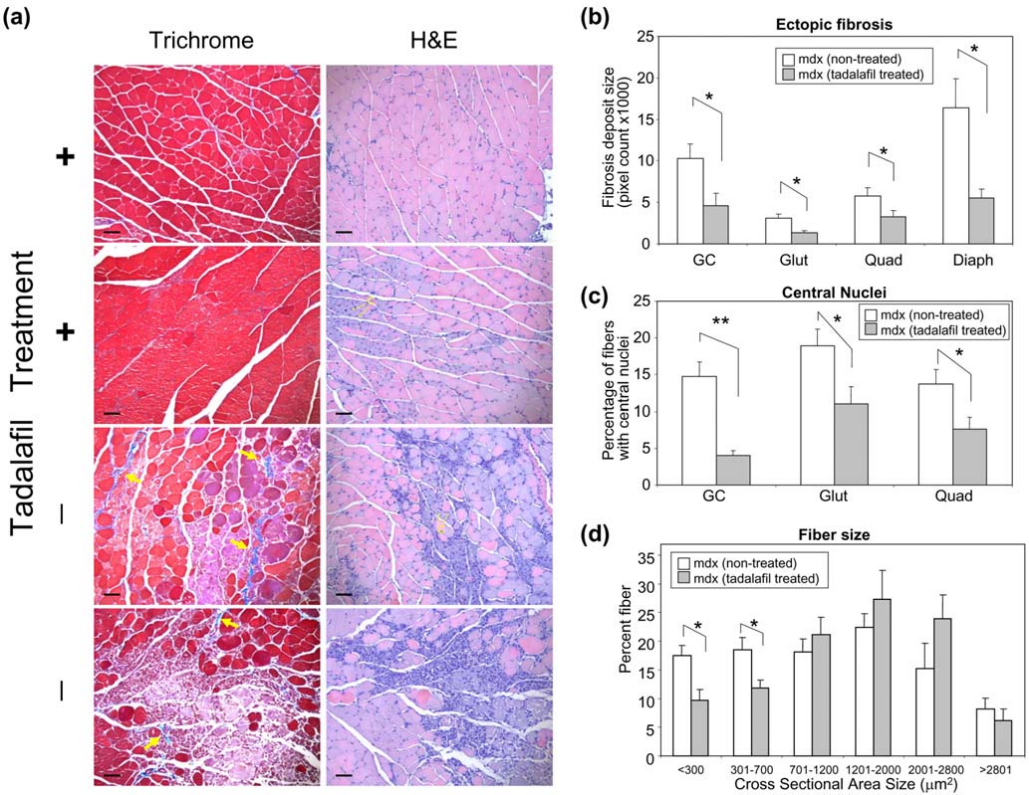
Evaluation of the efficacy of tadalafil by conventional histology. (a) Two images from different animals in tadalafil-treated and non-treated groups are presented for trichrome staining of gastrocnemius muscles (left panels): Ectopic fibrosis in extensively damaged areas was observed in the non-treated group (−) (yellow arrows). In the treatment group (+), there are still damaged fibers and ectopic fibrosis observed, but not to the level seen in the non-treated group. In H&E staining of gastrocnemius muscles (right panels), non-treated *mdx* mice showed many basophilic (purple) small cells reminiscent of infiltrating cells, proliferating fibroblasts/myoblasts, and tadalafil-treated group showed less. Myofibers with central nuclei, suggestive of regenerating fibers, are basophilic and prominent in the non-treated group, but were also observed in the treated group. Heterogeneity of fiber size was more prominent in the non-treated group. Yellow diamond arrows with “C” point to myofibers with central nuclei. Black scale bars on each micrograph image represent 50 µm. (b) The area size for ectopic fibrosis with blue staining was calculated in pixel size (n = 6 and 5 for non-treatment and tadalafil treatment groups). Tadalafil significantly reduced the amount of fibrosis in gastrocnemius (GC), gluteus (Glut), quadriceps (Quad), and diaphragm (Diaph) muscles (*: p<0.05 by t-test). (c) The percentage of myofibers with central nuclei is shown in a histogram. Tadalafil treatment reduced the percentage of central nuclei (*:p<0.05, **:p<0.01 by t-test) (d) The variation in fiber size in gastrocnemius muscles was quantified and shown as percentage fiber distribution. In non-treated *mdx* mice, the fiber size was uneven ranging from small (<300 µm^2^) to large (>2800 µm^2^) cross sectional area. Tadalafil treatment reduced the variation by lowering the percentage of small myofibers (*: p<0.05 by t-test)

To test the hypotheses that (i) preventing myofiber degeneration can impede the progress of muscular dystrophy and that (ii) agents targeted towards the NO-cGMP pathway can be used as therapeutic candidates, *mdx* mice were treated with orally administered tadalafil, a phosphodiesterase-5 inhibitor (PDE5I). This drug increases intracellular levels of cGMP in the vascular smooth muscle cells, causes vasodilation and increases blood flow to the target organ. Without treatment, *mdx* mice showed an increased amount of damaged myofibers in hindlimb and diaphragm as revealed by their blue color after Evans Blue dye injection (Figure 7a, top 6 panels, and Figure 7b, top 3 panels). When *mdx* mice were treated with tadalafil, the amount of dying fibers was decreased (Figure 7a, bottom 6 panels and Figure 7b, bottom 3 panels). When hindlimb muscles and diaphragm of *mdx* mice were sectioned and evaluated under fluorescence microscopy, *mdx* mice without treatment showed an increased red-fluorescent staining while exposure with tadalafil diminished the injury in the gastrocnemius, gluteus maximus, quadriceps, and diaphragm muscles (Figure 7c&7d). To further confirm this finding, conventional histology with trichrome and hematoxylin-eosin (H&E) staining (Figure 8a) was performed on both tadalafil-treated and non-treated *mdx* mice. The extensive myofiber damage and ectopic fibrosis (Figure 8a, blue deposit in the interstitial area in trichrome staining, yellow arrows) was prominent in non-treated *mdx* mice. The extent of ectopic fibrosis was decreased by tadalafil treatment (Figure 8b for quantification, p<0.05). The numbers of myofibers with central nuclei was reduced by tadalafil treatment (Figure 8c). The variation in fiber size prominent in non-treated *mdx* mice, was reduced by tadalafil treatment (Figure 8d, p<0.05). These data suggest that myofiber protective effect by tadalafil can slow down the progress of the disease.

## Discussion

### Functional Ischemia is likely an essential cause of muscular dystrophy

The still unsettled debate over the role of blood flow regulation in DMD was initiated as early as the mid-19th century. The most important question remaining is whether disturbed circulation in skeletal muscles plays a primary role in the pathogenesis of DMD. Using newly established assays to follow *in vivo* myofiber cell death, combined with microcirculation analyses, we have documented evidence that supports the essential role of vasoregulation in the pathophysiology of the skeletal muscles in *mdx* mice and have shown that therapeutic drugs targeted towards vasoregulation are effective in decreasing myofiber damage in these mice. This finding is consistent with previous reports that transgene overexpression of nNOS in *mdx* mice [41], [42] or dystrophin in their vascular smooth muscle cells [43] ameliorated the muscle damage. The transgenic expression studies of nNOS in *mdx* mice utilized a skeletal muscle specific promoter [41], [42], and provided the basis for an assertion that nNOS protects myofibers by directly working on myofibers. However, NO is a secretable molecule, and increasing evidence support that NO is utilized in intercellular communication [44], [45]. Thus, the previous studies have not excluded the possibility that NO produced from myofibers protect myofibers by acting on vascular regulation. Past reports support that nNOS expression in myofibers control the vasodilation in skeletal muscles [8].

The “functional ischemia” theory of muscular dystrophy demonstrated that under the stress of vasoconstrictors, the patients' muscles are unable to increase blood flow back to normal even after muscle contraction [4], [5]. A major innovation and strength of this current investigation measuring the peripheral RBC flux is its ability to reveal functional ischemia by a simpler scheme of stimulation than those utilized in previous macroscopic studies, enabling simple and quantitative discussions (Figure S7, supporting information).

Until now, one of the technical limitations of cell death studies came from the fact that myofiber cell death is always increased in the muscles of muscular dystrophy subjects (see Figures 7 abc&d). With conventional assays, there is considerable amount of cell death even before pharmacological/molecular interventions are applied, masking the effect by interventions even if they induce further cell damage. Our new method of myofiber cell death eliminated the already dead cell population by using a membrane potential-dependent dye, DiOC_6_, which is incorporated only by live cells at the beginning of experiment. This new approach enabled quantification of the specific effect of physiopharmacological interventions and allowed simple and clear discussions.

By using direct observation of the live animals, we documented a complete lack of the stimulated production of two vasodilative molecules, NO and H_2_O_2_, in response to muscle contraction: a possible cause of functional ischemia. Detailed observation detected a slightly elevated basal level of NO production in *mdx* mice, and its release from non-myofiber type cells (Figure 2). Since it is known that the sarcolemmal expression of nNOS is lost in *mdx* mice [46], and activity [47] and expression level [4] of eNOS is unchanged in the cardiac and skeletal muscles of *mdx* mice or DMD patients, the high basal level of NO is likely from upregulated expression of inducible NOS (iNOS) [48]. Further experiments are required to confirm this notion. By replenishing NO to the myofibers of *mdx* mice, blood flow to muscle was improved and contraction-induced myofiber damage was prevented (Figure 4). The same finding was confirmed by application of clenbuterol or 8-CPT cGMP. Myofiber protection by 8-CPT cGMP is likely through a vascular control, because its benefit was completely abolished by further addition of ATII at the vasoconstrictive dose. This inhibitory effect of ATII is likely through vascular regulation, but not its direct catabolic function on myofibers, because ATII alone did not cause myofiber damage. These experiments suggested the primary role of functional ischemia in contraction-induced myofiber damage in *mdx* mice. Functional ischemia is necessary as a cause of contraction-induced myofiber damage.

### Possible involvement of the other vasodilator, EDHF

Previous studies on EDHF were predominantly performed on vascular systems and to a lesser extent on skeletal myocytes. There have been many confounding opinions as to the identity of EDHF, its generation site, its action site, its existence as (a) secretable molecule(s), and involvement of gap-junctions, to name a few, leading to our putative understanding that EDHF is a heterogenic entity of vasodilative molecules/factors whose behavior is variable depending on the tissues of study. The action site of apamin and charybdotoxin, two well-established pharmacological inhibitors of EDHF, has also been controversial until recently. Previously, they were considered to work at the very downstream of EDHF function by inhibiting the hyperpolarization of vascular smooth muscle cells. In *ex vivo* vascular experiments, however, increasing evidence [34], [35] suggests that apamin and charybdotoxin work on endothelial cells (upstream), rather than on vascular smooth muscle cells (downstream). Our finding suggests that skeletal myocytes may be another upstream action site of these inhibitors. EDHF, in concert with NO, may function to convey signals for musculo-vascular communication, though whether the detected H_2_O_2_ is *an* EDHF has to be confirmed.

The reason for the prominent production of fluorescence signals from non-myocyte (non-fiber-like, dotty staining) only in assays for NO (Figure 2a, the first and second columns, arrows) but not in those for H_2_O_2_ (the third and fourth columns) may be in part because infiltrating cells like macrophages express iNOS upon differentiation [49] while losing enzymes for H_2_O_2_ production [50], [51] and enriching its scavengers including catalase and glutathione peroxidase [52].

### Quantitative evidence of the “two hit” mechanisms is provided by comparison of two animal models: functional ischemia model in mdx mice vs. severe ischemia model in control mice

The quantitative comparison of control and *mdx* mice showed that inhibition of NO/EDHF alone caused functional ischemia comparable to that of *mdx* mice (Figure 6), but did not induce muscle cell death to the same extent as was seen in *mdx* mice. To induce contraction-dependent myofiber damage in control mice to the comparable extent of that of *mdx* mice, a more severe ischemia and more strenuous tetanic stimuli were necessary (Figure 5 and 6). These results suggest that independent of abnormal blood flow response, myofibers in *mdx* mice are already vulnerable to mechanical stress. Our experiments have demonstrated the existence of at least two causes leading to contraction-induced myofiber damage in *mdx* mice: (i) a pharmacologically treatable factor (RBC flux), which is mediated by NO/EDHF and possibly other molecules, and (ii) elements independent of NO/EDHF or blood flow regulation (supporting information Figure S8a–d). These two factors may explain the previously suggested “two-hit” mechanism in this disease [23]. Neither one of these two factors alone causes muscle damage to the level observed in *mdx* mice. Only when the above two factors are affected (“hit”) at the molecular or pathophysiological level, does the muscle develop significant amount of damage.

Despite the result showing that inhibition of only NO production is not enough to cause cell death (Figure 5), it is intriguing that addition of SNAP (an NO donor) can prevent contraction-induced cell death (Figure 4). Perhaps mammals have evolved so that there are redundant factors provided for muscle contraction-induced blood flow increase and protection from contraction-induced stress. Thus, even if EDHF synthesis/function fails, another factor such as NO may substitute in its absence and vice versa. Replenishing NO can, by itself, normalize the blood flow and prevent ischemia-induced injury despite the possibility that the affected muscles are still inherently susceptible to contraction-induced damage.

### PDE5 Inhibitors can be a potential therapeutic agent to treat DMD

Additional support linking vasoregulation to the cause of muscular dystrophy was demonstrated by the effective treatment of the *mdx* mice with a PDE5 inhibitor, tadalafil. This data is consistent with the experiments from *in vivo* microscopy showing the essential role of functional ischemia in the pathogenesis of muscular dystrophy, and it is expected that this drug as well as other vasoregulatory molecules can be a future therapeutic target of this disease. Although vasoactive agents (NO, 8CPT-cGMP, and clenbuterol) almost completely prevented myofiber damage in our short-term experiments from *in vivo* microscopy (Figure 4), there was still remaining myofiber damage observed in some animals treated with tadalafil (Figure 7a and 7b). This may be due to a downregulation of the effect of the drug after weeks of treatment, or because of possible variations in the extent of *ad lib* movements of individual animals that will require further investigation. In this study, we prioritized biological question about the efficacy of tadalafil and started the drug treatment before birth and continued during lactation and weaning until 4 weeks of age, based on the fact that myofiber damage in always upregulated in *mdx* mice and DMD patients even before birth [53], [54] and that this drug has high transplacental distribution capability and is lactationally secreted [55]. Biologically, our data from experiments in tadalafil treatment supports that histological changes are due to the increased turnover of muscle degeneration and regeneration. The numbers of myofibers with central nuclei, characteristic of active regeneration was reduced by tadalafil treatment. This observation suggests that PDE5I can ameliorate the progress of the disease mainly through its myofiber protective function, but not through upregulation of regeneration and that regeneration of myofibers often observed in *mdx* mice may occur secondarily to upregulated muscle degeneration. To fully establish its therapeutic potential of tadalafil treatment, however, long-term therapeutic study on post-weaning *mdx* mice is required.

Our data suggest that the myofiber protective effect of cGMP in the acute phase experiment is likely through its vascular role, but do not exclude the possibility that the NO-cGMP pathway exerts anabolic effect through a non-vascular mechanism in more chronic way. More specific experiments with molecular biological interventions are in progress to further examine the pathophysiology of DMD and test the long-term efficacy of PDE5I on adult *mdx* mice. Furthermore, our conclusion of the primary role of vascular role in the pathogenesis of muscular dystrophy does not deny or exclude the possibility of the currently accepted theory of “membrane vulnerability”. In fact, membrane vulnerability may be the other factor of the “two-hit” mechanism.

### Conclusion

In summary, we have documented strong evidence for the primary role of functional ischemia in the pathogenesis of muscular dystrophy. We have for the first time quantitatively demonstrated the existence of the “two-hit” mechanism in this disease. Importantly, a promising therapeutic approach was demonstrated with a vasoactive drug.

## Materials and Methods

### Reagents

Krebs Ringer (“KR”, d-glucose 1.8 g/l, MgCl_2_ 46.8 mg/l, KCl 340 mg/l, NaCl 7 g/l, Na_2_HPO_4_ 100 mg/l, NaH_2_PO_4_ 180 mg/l, NaHCO_3_ 1.26g/l, pH = 7.30), and PBS (phosphate buffered saline, NaCl 8 g/l, KCl 200 mg/l, Na_2_HPO_4_ 1.44 g/l, KH_2_PO_4_ 240 mg/l, pH = 7.4) were prepared fresh and pH adjusted for each experiment. L-NAME (Sigma, *N*ω-Nitro-L-arginine methyl ester, 1 mg/ml), charybdotoxin (Sigma, 0.1 µM), apamin (Sigma, 1 µM), α-bungarotoxin (Invitrogen, 1 µg/ml), DiOC_6_ (3,3′-dihexyloxacarbocyanine iodide, Invitrogen, 80 nM), Annexin-V AlexaFluor 350 (Invitrogen), SNAP (Sigma, S-nitroso-N-acetylpenicillamine, 100 µM), 8-(4-chlorophenylthio)-guanosine 3′, 5′-cyclic monophosphate (8-CPT cGMP, Sigma, 500 µM), clenbuterol (Sigma, 0.05 mg/ml), and angiotensin-II (Sigma, 1 µg/ml) were applied in KR as a vehicle. Evans Blue dye (Sigma, 5 µl/gBW of 10 mg/ml solution in PBS) and tadalafil (Eli Lily and Company, 1 mg/100 ml water) were used for PDE5I experiments.

### Mouse Strains and *in Vivo* Microscopic Observation of Skeletal Muscles

An *mdx* mouse colony was established by mating hemizygote male and homozygote female of *mdx* strain (*C57BL/10ScSn-Dmd^mdx^/J*) purchased from Jackson Laboratories. Control mice were inbred *C57BL/10ScSn*. Adult male mice between the age of 3 months and 6 months were used in this study except for the PDE5I experiment. A previous study that showed DMD involves a pathologic change of sternocleidomastoid muscles in humans [56] was the basis for our experiments to investigate the pathophysiological changes in the corresponding mouse sternomastoid muscle. All the procedures related to animal experiments were reviewed and approved by the Subcommittee on Research Animal Care of Massachusetts General Hospital. We followed Jeff W. Lichtman's method [57] with modifications. Mice were anesthetized by intraperitoneal injection of pentobarbital (50 mg/kgBW), shaved on the front neck, intubated with a 20 gage polyethylene tube, mechanically ventilated, and warmed at 37°C on a Kapton sheet heater. The right and left sternomastoid muscles were exposed aseptically and superfused with sterile Krebs Ringer. Water-dipping objective lenses were utilized for live epifluorescent observation. An intensified SIT Camera (Hamamatsu Photonics, C2400) was installed on a Nikon Eclipse-800 microscope to capture the low-intensity signal at the real-time video rate (30fps). A conventional CCD Camera (SPOT, Diagnostics Instruments, Inc.) allowed documentation of high resolution fluorescence images. Fluorophores and the filter sets utilized were DiOC_6_ (484/501 nm for excitation/emission hereafter), Hoechst 33258, (352/461), Propidium Iodine (536/617), and Alexa Fluor 594-conjugated α-bungarotoxin (590/617). An automated z-axis focusing system (Prior, Proscan II) was used for acquiring images on focus planes at different depth. To obtain qualitative morphological documentation, two-dimensional pictures were derived from stacks of photographs by extracting in-focus information from z-axis planes to create image mosaics by MetaMorph (Molecular Devices). Quantitative analyses for fluorescence intensity were performed on single images documented using the CCD camera.

### Muscle Stimulation

An electrical pulse was generated using a Peripheral Nerve Stimulator (Innervator 252, Fisher&Paykel Healthcare), and delivered to the muscle surface via a coated stainless probe at supramaximum condition (see supporting information, Figure S2). A minus probe end (ground) was placed on the proximal part of muscle and a plus end (source) was placed on the distal muscle area. The experimental design is based on either a single train of stimuli (5 seconds of 50Hz, for RBC flux experiment), or a series of trains of stimuli (a repeated pattern of 5 seconds of 50Hz with 30-second intervals, for NO/H_2_O_2_ production, cell death experiments). The electrical current did not cause direct damage on the vicinity of contact area. Pharmacological reagents at physiological concentrations described above were applied directly to the area.

### Blood Flow Analysis

#### Labeling RBCs and Injection

Mouse RBCs, prepared from the inbred allogenic mice with heparinization, were washed in KR, and incubated with 2 µM PKH26GL (Sigma) for 5min at room temperature. After staining, the remaining dye was quenched with heat-inactivated serum, and washed away with KR. A bolus of 50 µl of a 12% haematocrit of the stained RBCs diluted in 37°C KR was injected into the penile vein. Using *in vivo* microscopy, video images of the circulating fluorescent-labeled RBCs (551/567 nm) were recorded on a DVD through the SIT video camera at 30 frames per second at the level of primary arterioles (Strahler's numbering system: see supporting information, Figure S1) [58]. No hemolytic response due to transfusion was observed during the time of observation up to 6 hours. After injection of the stained RBCs, animals were kept quiet and stable for 30 minutes. The average baseline RBC flux was recorded and calculated from 5 minutes' flux measurement. Reagents were applied at this point. A 10 minute recording was made after muscle contraction by one tetanic stimulus as described above. At the end of the experiment, mouse whole blood was obtained from the heart. The whole RBC concentration and the ratio of stained/non-stained RBCs were counted using a hematocytometer.

#### Analyses of RBC Flux

Recorded DVD video images were transferred to Video Savant™ image files through a frame grabber (MV-1000, MuTech). Video images were reviewed on a monitor in adjusted speed in order to visualize individual stained RBCs. RBC flux was measured by counting labeled RBCs flowing through a focused primary arteriole per minute. One arteriole per mouse was selected from NMJ or non-NMJ area. The absolute numbers of RBC flux was calculated from the ratio of stained/non-stained RBCs. The total increase of RBC flux (for Figure 6) was calculated as the integral of the percent increase of RBC flux, or the area size of the area bound between the curve of RBC flux and basal line (100 percent line), shown as the green area subtracted by red area in the supporting information, Figure S6.

#### Vascular Oppression

To impose a severe ischemic stress on the muscle, vascular oppression was applied from backside (dorsal side) by pressing the muscle with 10 g force using a smooth double-rod of a 0.3 mm diameter. The applied force of 10g was measured by a force-displacement transducer (FT03C, Grass Instruments) connected to an AC/DC strain gage amplifier (P122, Grass Instruments) and recorded by a computer software (“Polyview”, Grass Instruments). This manipulation did not cause any detrimental effect by itself or in combination with tetanic stimulation (*see above for results and* *Figure 5*). Measurement of RBC flux under this condition confirmed the decreased blood flow but not a complete blood vessel obstruction (*see above for results and* *Figure 6*).

### Measurement of *In Vivo* NO and H_2_O_2_ Production

The membrane permeable dye for nitric oxide (NO) detection, DAF-FM (4-amino-5-methylamino-2′,7′-difluorofluorescein, Invitrogen), was applied at 1 µM onto the observation area of the sternomastoid muscle and incubated for 15 minutes at 37°C. DAF-FM becomes fluorescent (excitation 495 nm, emission 515 nm) when it couples with NO in physiological conditions. Baseline fluorescence intensity was measured before the muscle stimulation. Five second tetanic stimuli were given to the muscle 6 times with 30 seconds intervals. After the stimulation, an *in vivo* fluorescence signal was acquired repeatedly every 10 minutes with a CCD camera and analyzed by an image software (MetaMorph, Molecular Devices). Fluorescent intensity was quantified by densitometry of the captured images using a standard fluorescent-intensity curve. Photo-activation and photo-bleaching of dye were avoided by limiting the exposure time to less than 1 second and using appropriate ND filters. For measurement of *in vivo* H_2_O_2_ production, membrane permeable carboxy-H_2_-DCFDA (2′,7′- dichloro- dihydrofluorescein diacetate, excitation 493 nm, emission 522 nm, Invitrogen) was applied at 5 µM and incubated for 15 minutes at 37°C. Five second tetanic stimuli were given to the muscle 12 times with 30 seconds intervals. Every 20 minutes after stimulation, an *in vivo* fluorescence signal was acquired.

#### Fluorescent light standard

Fluorescent microspheres (LinearFlow™ Green Flow Cytometry Intensity Calibration Kit, Molecular Probes, 6 µm for 488 nm excitation/515 nm emission) were used as the internal standard to normalize the fluorescence intensity. In order to generate a standard fluorescent-intensity curve, three different types of microspheres (Component C, D, E; 0.4%, 2%, 10% each, relative intensity of Component F; 100%) were selected and average fluorescent intensities per pixel were calculated for each component. Exposure times and gains were determined in order for each dye to fit within a linear range of this standard curve and maintained throughout the experiment.

### Real-Time *In Vivo* Monitoring of Muscle Cell Death

#### Staining and Microscopy

Sternomastoid muscles were stained with α-bungarotoxin (BTX) and DiOC_6_. Postsynaptic acetylcholine receptors were labeled with 1 µg/ml of Alexa Fluor 594-conjugated α-BTX for 10 minutes. According to previous studies, this dosage did not block postsynaptic activity. DiOC_6_ (80nM, 15 min) stains mitochondria and endoplasmic reticulum (M/ER) only in live cells. At this concentration, DiOC_6_ staining was not cytotoxic to skeletal muscle cells. DiOC_6_ yielded irreversible staining and remained on their specific target until they were removed by the biological turn-over or underwent fluorescence bleaching. Thus, documentation of the chronological alteration in the morphology of neuromuscular junctions (NMJs) and of organelles was possible. Specific tissue sites were identified and followed using the shape and location of NMJs as signposts. In *mdx* mice, a 5 second tetanic stimulation (TS) was applied 6 times as described above. In control mice the TS was applied 6 or 12 times and compared. Stained muscles were recorded at 30 minutes, 2 hours, 4 hours and 6 hours after stimulation. Video images were recorded using an intensified SIT camera at 30 frames per second, recorded on DVD, and the numbers of dead myofiber loci in the entire muscle were counted manually.

#### Fiber Loci Counting and Criteria for Myofiber Damage

We counted between 70 and 80 myofibers per scan for both control and *mdx* mice. These numbers are similar to but slightly less than those of fiber counts in the original method on sternomastoid muscles [59], because we observed the few superficial layers of the muscles due to the nature of our staining. To scan the whole area, the microscopic image field was moved transversely (along x-axis) back and forth, shifting along y-axis with each scan (supporting information, Figure S4a). For the feasibility of counting, fibers in different image fields of observation were regarded as different. Thus, scan #1 (supporting information, Figure S4a) counted fibers from 1 to 70, scan #2 counted from 71 to 140, and so forth. The grand total numbers of counted fibers of 350 to 400 (70 to 80 multiplied by the numbers of 5 transverse scan) covered the actual area size of approximately 10 mm^2^ and were consistent throughout the experiment, both for control and for *mdx* mice. Because of the anatomical structure of the sternomastoid muscle, the distal (mastoid) end of the muscle tissue was not counted. Granulated, rippled, or bulged cluster of staining with DiOC_6_ were considered as criteria to define abnormal distribution of M/ER of damaged fibers. Myofibers manifesting any of these traits eventually led to regional cell death, as detected by the dye exclusion staining method using propidium iodide (1 µg/ml, 15 min) or Hoechst 33258 (2 µg/ml, 3 min) and Annexin-V AlexaFluor 350 staining (30 min). By our criteria, granular, rippled, and bulged areas within a single fiber were counted as one damaged myofiber locus as long as the lesion was continuous (supporting information, Figure S4b). If any two lesions were separated by an intact area with a normally striated pattern extending into different field of observation, those were counted separately. If two lesions were not continuous, and were separated by an area where staining was completely lost, or the fiber was severed, resulting in different fields of observation, those were regarded as different loci.

### Tadalafil Treatment and Evans Blue Assay

To achieve oral administration of tadalafil, *mdx* mice were treated with 1mg tadalafil/100 ml drinking water from the beginning of pregnancy. The drug containing water was changed twice per week. For histological detection of damaged myofibers, we followed a previously described method [60] with a minor modification. At 4 weeks after birth, Evans Blue dye was injected intraperitoneally (50 µg/gBW in PBS) to detect and quantify the amount of damaged myofibers. After 6 hours, mice were sacrificed and skeletal muscle tissues harvested. Muscles were pin-stretched and frozen in OCT (Tissue-Tek) at −80°C. The harvested muscles were cryosectioned at 7 µm, acetone fixed for 10 minutes at −20°C, and observed under fluorescence microscopy (540/610nm).

### Trichrome and Hematoxylin-Eosin (H&E) Staining

*Mdx* mice are anesthetized, heparinized, blood removed and perfuse-fixed by cardiac injection of 4% paraformaldehyde (PFA) freshly prepared in PBS. Hindlimb muscles (gastrocnemius, quadriceps, and gluteus) were harvested and further fixed by immersing in 4% PFA in PBS for 24 hours before being embedded in paraffin and sectioned at 4 µm thickness using a rotary microtome (Leica). Sections were stained by standard hematoxylin-eosin (H&E) and trichrome staining.methods. Briefly, for H&E staining, deparaffinized and hydrated sections were treated in Harris-modified hematoxylin with acetic acid solution (Fisher), washed in water and counterstained with saturated eosin-Y solution (Richard Allan Scientific) for 2 minutes respectively. For Masson trichrome staining, the sections were stained by following the manufacturer's instructions (Sigma). Ectopic fibrosis was determined by the specific blue color deposition from trichrome staining followed by image analysis with Metamorph on cross sections (gastrocnemius, gluteus, quadriceps) or longitudinal sections (diaphragm). We excluded areas for tendons, normal epimysiums, perimysiums and endomysiums as well as metachromatic myofibers (presumably unhealthy myofibers). 10 to 20 random areas are chosen from 3 different sections. Pixel count for fibrosis per field of view (1315×1033 total pixels) was measured by averaging the values obtained. The cross sectional area size (µm^2^) was measured on H&E stained images by Metamorph and the distribution of fiber percentage was calculated. The numbers of myofibers with central nuclei are counted based on H&E staining of cross-section (gastrocnemius, gluteus, and quadriceps). Infiltrating cells and satellite cells are excluded.

### Statistics

The normality of the data was evaluated by the Kolmogorov-Smirnov test. The comparison of means was performed with one-way ANOVA or t-test for multiple/two group comparisons. The increment from basal level in blood flow or cell death was assessed by one-way repeated measures ANOVA. Medians were also compared by Mann-Whitney U test. For all tests significance was accepted when P<0.05.

## Supporting Information

Figure S1 Schematic drawing of arteries, arterioles, venules and veins running through the right sternomastoid muscle of mice (equivalent of the medial part of the sternocleidomastoid muscle in humans). The drawings of 1° (primary) arterioles and capillaries are omitted for simplification. The measurement was made at locations enriched with neuromuscular junctions (shown as “NMJs”) and non-neuromuscular junctional areas (shown as “A”, “B”, and “C”). Nomenclature based on Strahler [58].
(2.27 MB TIF)

Figure S2 Comparison of muscle contractile force generated by 50Hz tetanic stimulation. Contractile force by the sternomastoid muscle was compared between control and *mdx* mice. Bipolar supramaximal electrical stimulation (20% beyond the voltage yielding maximal contractile force) was given directly onto sternomastoid muscles. The head of the mice was fixed by pinching the mastoid portion of the temporal bone. After the preload of 5 gram was stabilized, the tension force was measured by a transducer ligated to the sternum. Train of four (TOF, 2Hz) and tetanic stimulation (50Hz) were given. The left graph shows the kinetic of force generation (gram) plotted against time (seconds). Each color represents different animals. The maximum force generated by tetanic stimulation was not statistically different between control and *mdx* mice (right side bar graph). Standard errors are attached to the bar graph. “n.s.” stands for not statistically significant (Student-t, p = 0.60)
(6.54 MB TIF)

Figure S3 Confirmation of damaged fiber detection methods Sternomastoid myofibers are induced to cell death by the same stimulation used in Figure 5. Three different areas are shown. Healthy fibers with normal striated pattern of DiOC_6_ staining (fiber#1&3) are void of stating by propidium iodide (PI, red) or Annexin-V (blue). Damaged fiber loci (fiber#2,4,5&6) according to the criteria shown in Figure S4 are also stained by PI and Annexin, confirming fiber death.
(10.41 MB TIF)

Figure S4 Method for counting the damaged myofiber loci. Scanning procedure is shown in (a). The image field on the microscope was moved transversely (x-axis) starting from the head side (mastoid). After each scan, the field of view was shifted along the y-axis towards the chest side (sternum) to avoid overlap with the previous scan. This process was repeated five times to cover the entire sternomastoid muscle. Because of the anatomical structure of the muscle, the extreme end of the muscle on the head side (mastoid) was not visualized. (b) Criteria for counting the damaged myofiber loci are shown in (b). DiOC_6_ staining of muscle organelles (M/ER) with rippled, bulged, or granular pattern were considered damaged myofibers that eventually will lead to local cell death. If any two or three of these patterns were found in a single myofiber, those are counted as one damaged locus, as long as the lesion was continuous ((i) Continuous lesion). Some myofibers were affected by damages of multiple onsets. If any two lesions were separated by an intact area with normal striated pattern of M/ER extending into another field of view, those were counted as two loci ((ii) Multiple onset). If any two lesions were discontinuous, separated by loss of fiber staining or severing and isolated into different field of view, those were counted as two ((iii) Discontinuous).
(4.86 MB TIF)

Figure S5 Microscopic images of myofibers from *mdx* mice after tetanic stimulation with our without SNAP treatment and from control mice receiving tetanic stimulation and varying degree of ischemic stress. Fluorescence images of chronological changes in the morphology of intact and dying cells in the *mdx* mice are shown (400x, black scale bars at the bottom of images represent 10 mm). The NMJs were stained with red fluorescence as signposts to detect individual fibers. Mitochondria and endoplasmic reticulum (M/ER) of sternomastoid muscles were stained by DiOC_6_. Clusters of these stained M/ER form a transverse striated pattern in the normal intact myofibers (green staining at 30 minutes). (a) In *mdx* mice, repeated tetanic stimuli (5 second stimulus at 50Hz, repeated 6 times with a 30 seconds interval) caused dying myofibers with an abnormal distribution of the DiOC_6_-stained components (a, Row1, Mdx+tetanic stimuli). At 4 hours, the M/ER showed a granular pattern of distribution in the damaged fiber loci (arrow heads). At 6 hours, those granular staining areas changed into a larger cluster of bright fluorescent spots to form a bulged structure (arrows). Administration of SNAP (NO donor, 100 mM) prevented the *mdx* mice fibers from undergoing contraction-induced damage (a, Row2, Mdx+tetanic stimuli+SNAP). (b) When L-NAME, apamin plus charybdotoxin, vascular oppression were all combined, tetanic stimulation caused myofiber damage in control mice (b, Row3). At 2 to 4 hours after stimulation, damaged myofibers exhibited a granular distribution pattern (arrow heads). Eventually, those damaged fibers showed bulged staining (arrows). When any of the following treatment was absent, myofiber damage did not occur to the similar extent seen in the group described above: L-NAME (b, Row4), apamin plus charybdotoxin (Row5), vascular oppression (b, Row6), tetanic stimulation (b, Row7). Tetanic stimulation and vascular oppression alone did not cause damage (b, Row8). Normal pretzel-like NMJ morphology in control mice (b, red staining) did not change into the abnormal fragmented shape seen in *mdx* mice (a) or vice versa, by any combination of treatments during the observation period.
(9.50 MB TIF)

Figure S6 Calculation of the total increase of RBC flux. Total increase of RBC flux is given by the integral of the curve from 0 to 10 minutes after tetanic stimulation for the percent from basal RBC flux. The formula is provided as *F*(n)+1/2*F*(10): from n = 1 to 9, where F(n) stands for RBC flux (%) minus 100 at time n (minute). Note that a complete embolization of arteries will theoretically result in the value of negative 950.
(2.37 MB TIF)

Figure S7 Illustration of functional ischemia by a conventional (macroscopic) and by a new (microscopic) approach. Contracting skeletal muscles require an increased blood flow to meet metabolic demands. Functional ischemia is a pathological state where this normal response is disturbed. (a) In order to observe functional ischemia, the previous studies measuring crude macroscopic blood flow required the muscles to be put under vasoconstrictive stress by sympathetic stimulants. Skeletal muscle contraction reverts the drop in the crude blood flow in normal muscles (a normal response of sympatholysis). Sympatholysis is absent in the affected muscles where normal vascular regulation mechanisms are lacking. Thus, normal muscles are rescued from ischemia by sympathetic vasoconstriction when superimposed by contractile stress but diseased muscles are put under the risk of continued ischemia. (b) In our new microscopic study, preloading with vasoconstrictive stress is unnecessary to observe functional ischemia. RBC flux increases after muscle contraction in normal subjects. This increase in RBC flux is perturbed in *mdx* mice. One of the technical advantages in analyzing RBC flux is to reveal a functional ischemia by a simpler scheme of stimulation than previous macroscopic studies. This observation is reasonable considering our understanding that the crude macroscopic blood flow measurement does not necessarily reflect peripheral (microscopic) RBC flux [61]. Note that RBC flux, RBC velocity, and vascular diameter are different parameters of microcirculation and may follow different kinetics, though all of these are important factors determining the state of local blood flow. We emphasize the importance of measuring the RBC flux in this type of analysis.
(2.57 MB TIF)

Figure S8 The relationship between functional ischemia and myofiber damage in control and *mdx* mouse models. (a) Normal blood flow response vs. lack of response in *mdx* mice. The results from our experiments in control and *mdx* mice supports the hypothesis that RBC flux in control mouse increases in response to muscle contraction via increased production of NO and H_2_O_2_, and *mdx* mice are deficient of NO and H_2_O_2_ production in response to muscle contraction resulting in functional ischemia and the risk for myofiber damage. (b) Quantitative analysis between the extent of functional ischemia and myofiber damage suggests that there are at least two major factors causing the contraction-induced myofiber damage in *mdx* mice: (1) functional ischemia, which is likely to be caused by the lack of NO/H_2_O_2_ production, and (2) a putative intrinsic factor which makes *mdx* myofibers inherently more vulnerable than those in control mice, independent of NO/H_2_O_2_ regulation. (c) *Mdx* mice under pharmacotherapy against functional ischemia are still inflicted by inherent weakness, but myofiber damage can be prevented. (d) Control mice inflicted with artificial ischemia do not show comparable amount of myofiber damage, because their myofibers are not inherently weak.
(4.16 MB TIF)
