# Supplementary figures and images for: Primary Role of Functional Ischemia, Quantitative Evidence for the Two-Hit Mechanism, and Phosphodiesterase-5 Inhibitor Therapy in Mouse Muscular Dystrophy
Source: PLoS One. 2007 Aug 29;2(8):e806. doi: 10.1371/journal.pone.0000806 (PMC1950086; doi:10.1371/journal.pone.0000806)

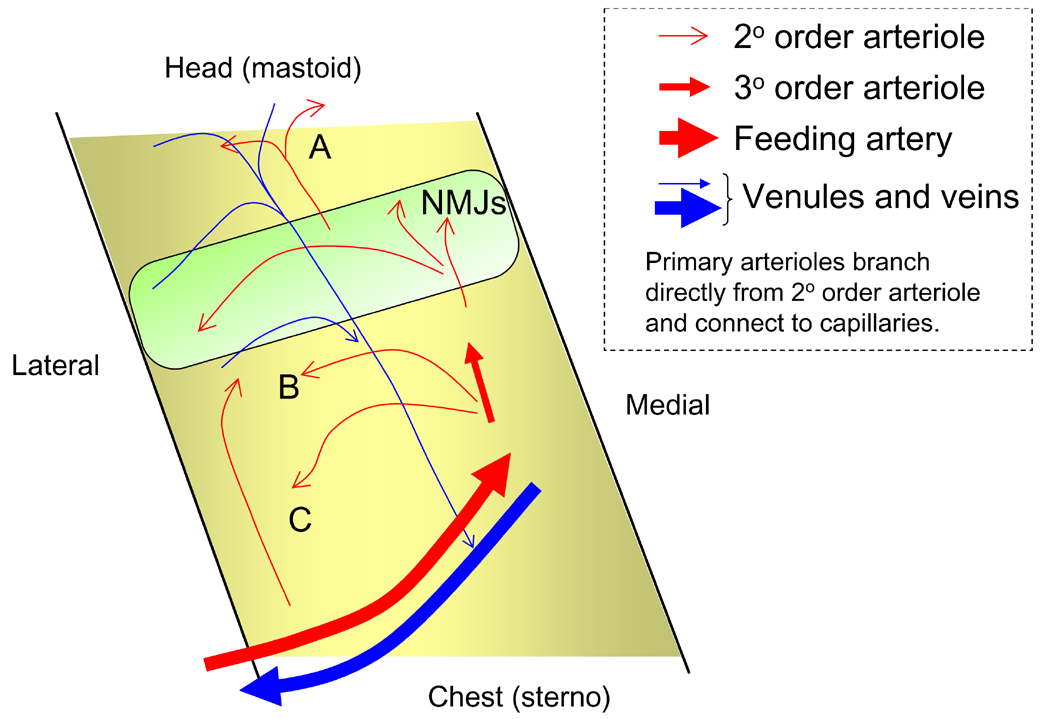

Supplement: Figure S1 — Schematic drawing of arteries, arterioles, venules and veins running through the right sternomastoid muscle of mice (equivalent of the medial part of the sternocleidomastoid muscle in humans). The drawings of 1° (primary) arterioles and capillaries are omitted for simplification. The measurement was made at locations enriched with neuromuscular junctions (shown as “NMJs”) and non-neuromuscular junctional areas (shown as “A”, “B”, and “C”). Nomenclature based on Strahler [58]. (2.27 MB TIF) [file pone.0000806.s001.tif]

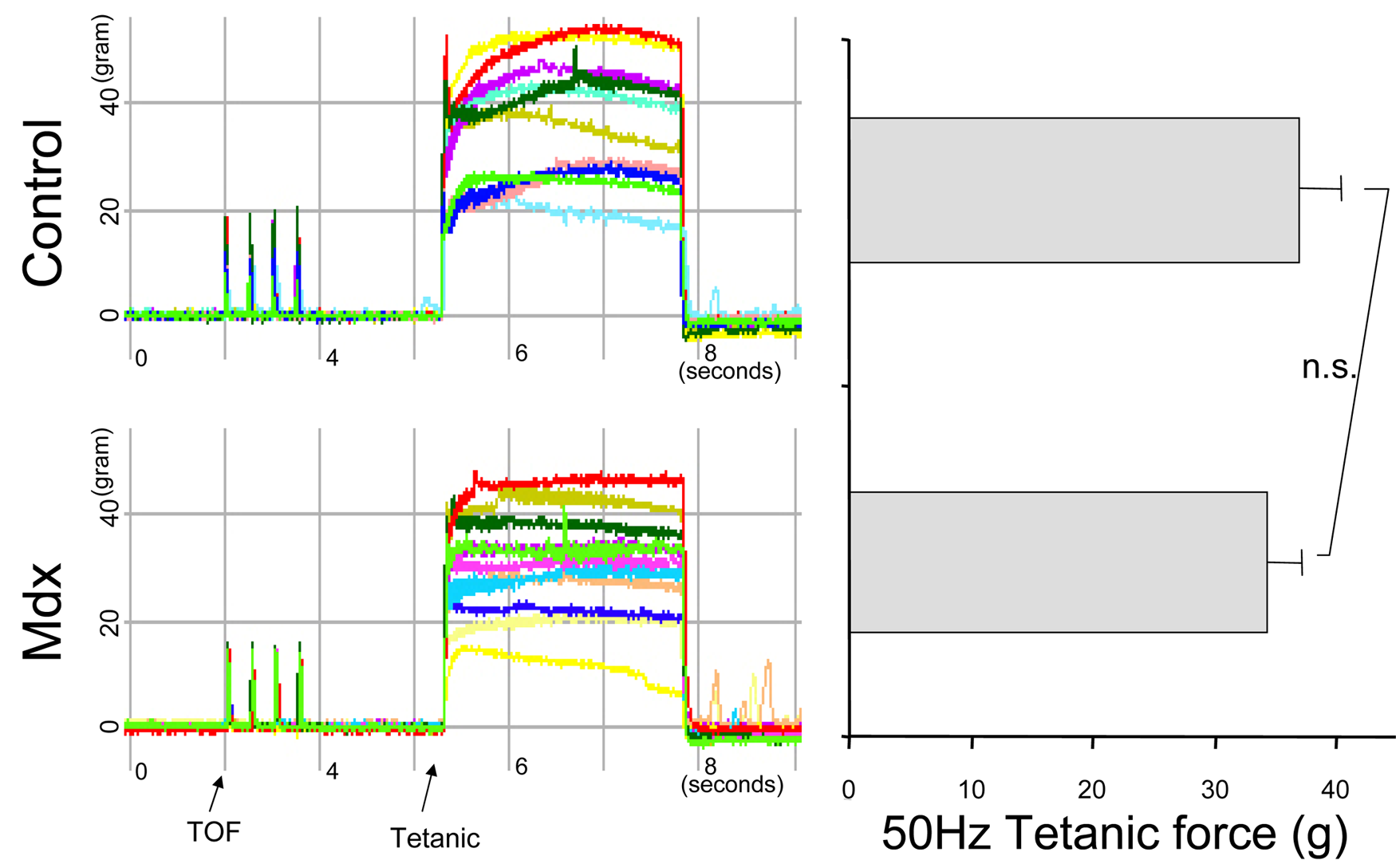

Supplement: Figure S2 — Comparison of muscle contractile force generated by 50Hz tetanic stimulation. Contractile force by the sternomastoid muscle was compared between control and mdx mice. Bipolar supramaximal electrical stimulation (20% beyond the voltage yielding maximal contractile force) was given directly onto sternomastoid muscles. The head of the mice was fixed by pinching the mastoid portion of the temporal bone. After the preload of 5 gram was stabilized, the tension force was measured by a transducer ligated to the sternum. Train of four (TOF, 2Hz) and tetanic stimulation (50Hz) were given. The left graph shows the kinetic of force generation (gram) plotted against time (seconds). Each color represents different animals. The maximum force generated by tetanic stimulation was not statistically different between control and mdx mice (right side bar graph). Standard errors are attached to the bar graph. “n.s.” stands for not statistically significant (Student-t, p = 0.60) (6.54 MB TIF) [file pone.0000806.s002.tif]

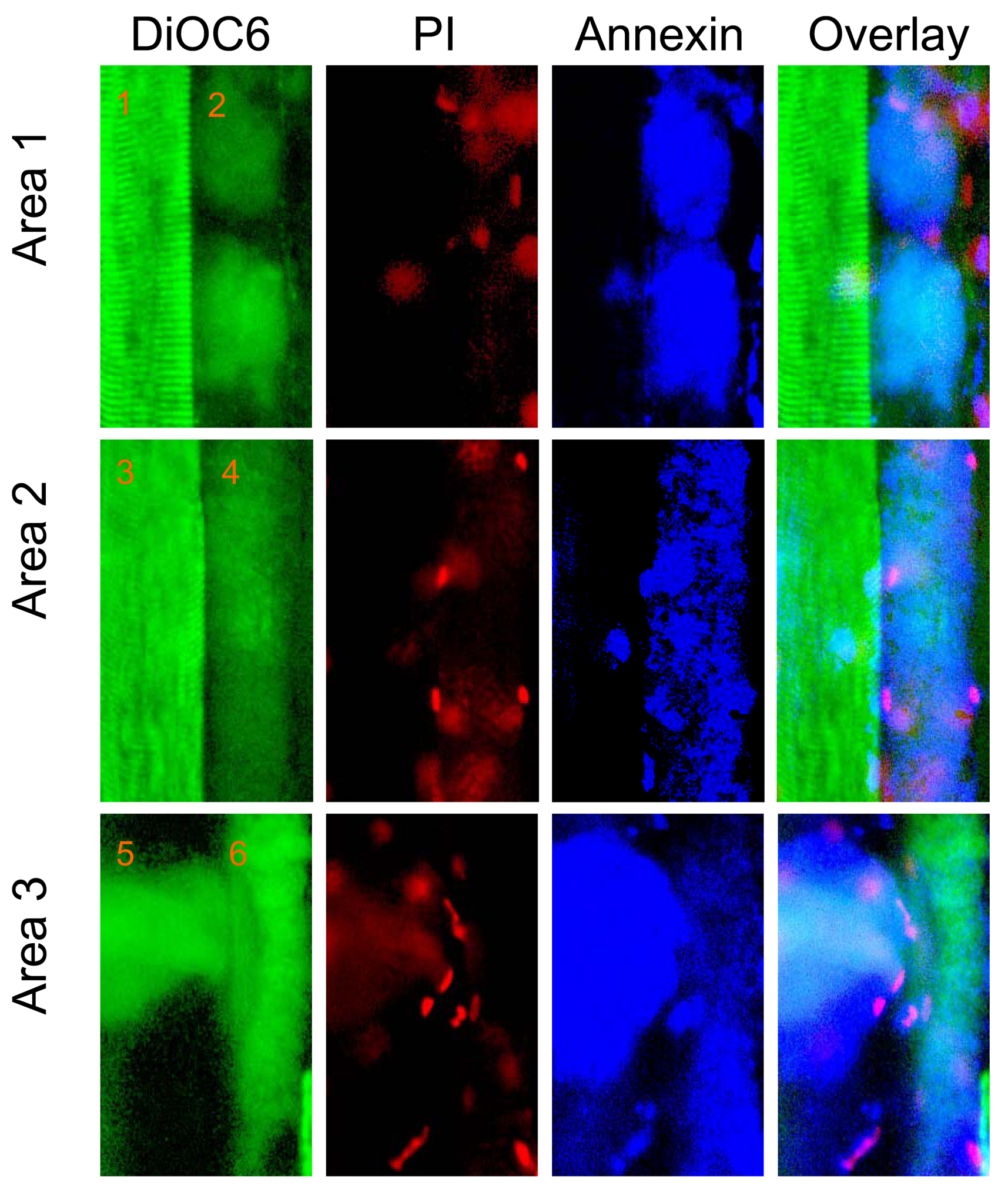

Supplement: Figure S3 — Confirmation of damaged fiber detection methods Sternomastoid myofibers are induced to cell death by the same stimulation used in Figure 5. Three different areas are shown. Healthy fibers with normal striated pattern of DiOC6 staining (fiber#1&3) are void of stating by propidium iodide (PI, red) or Annexin-V (blue). Damaged fiber loci (fiber#2,4,5&6) according to the criteria shown in Figure S4 are also stained by PI and Annexin, confirming fiber death. (10.41 MB TIF) [file pone.0000806.s003.tif]

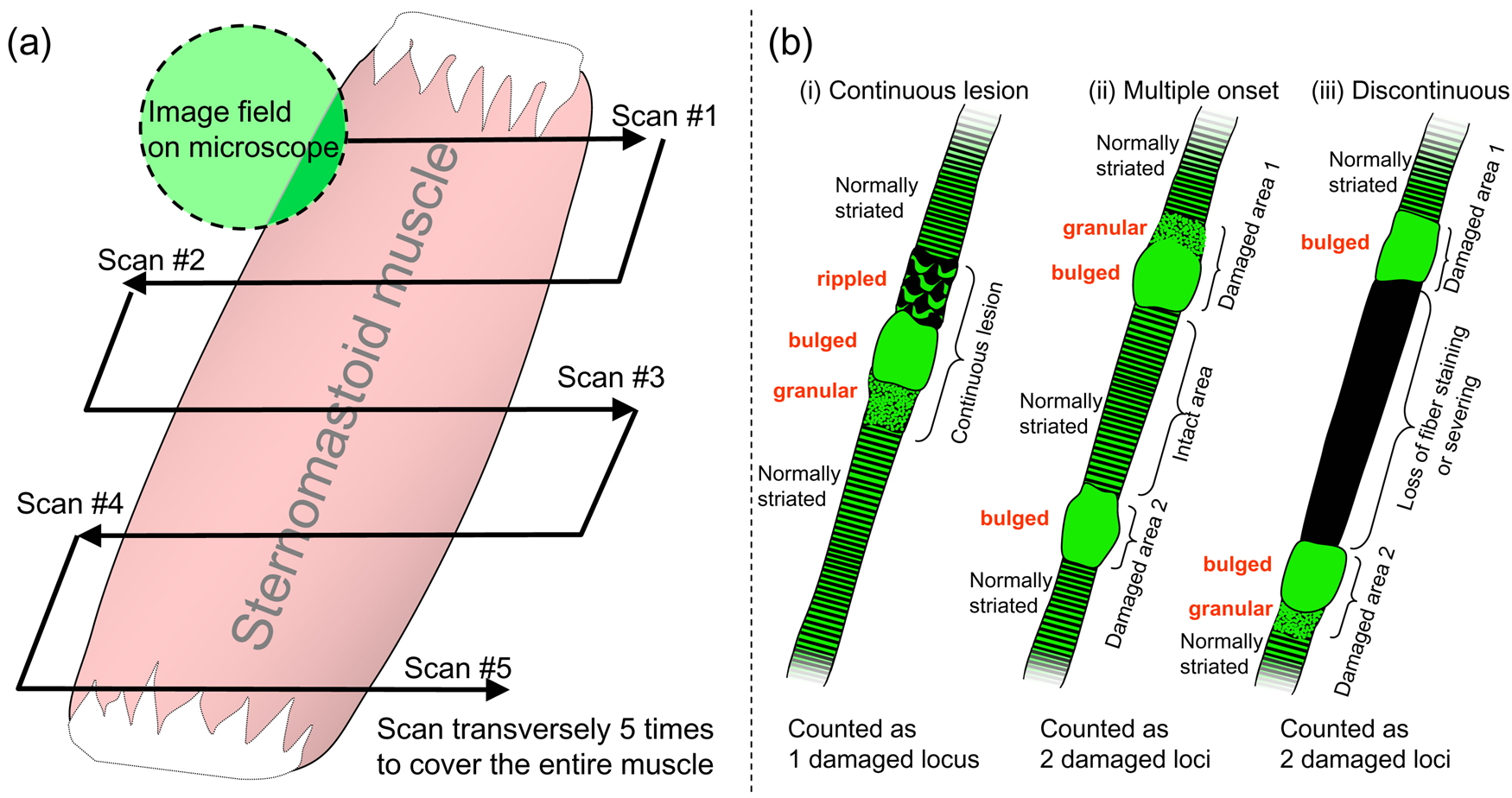

Supplement: Figure S4 — Method for counting the damaged myofiber loci. Scanning procedure is shown in (a). The image field on the microscope was moved transversely (x-axis) starting from the head side (mastoid). After each scan, the field of view was shifted along the y-axis towards the chest side (sternum) to avoid overlap with the previous scan. This process was repeated five times to cover the entire sternomastoid muscle. Because of the anatomical structure of the muscle, the extreme end of the muscle on the head side (mastoid) was not visualized. (b) Criteria for counting the damaged myofiber loci are shown in (b). DiOC6 staining of muscle organelles (M/ER) with rippled, bulged, or granular pattern were considered damaged myofibers that eventually will lead to local cell death. If any two or three of these patterns were found in a single myofiber, those are counted as one damaged locus, as long as the lesion was continuous ((i) Continuous lesion). Some myofibers were affected by damages of multiple onsets. If any two lesions were separated by an intact area with normal striated pattern of M/ER extending into another field of view, those were counted as two loci ((ii) Multiple onset). If any two lesions were discontinuous, separated by loss of fiber staining or severing and isolated into different field of view, those were counted as two ((iii) Discontinuous). (4.86 MB TIF) [file pone.0000806.s004.tif]

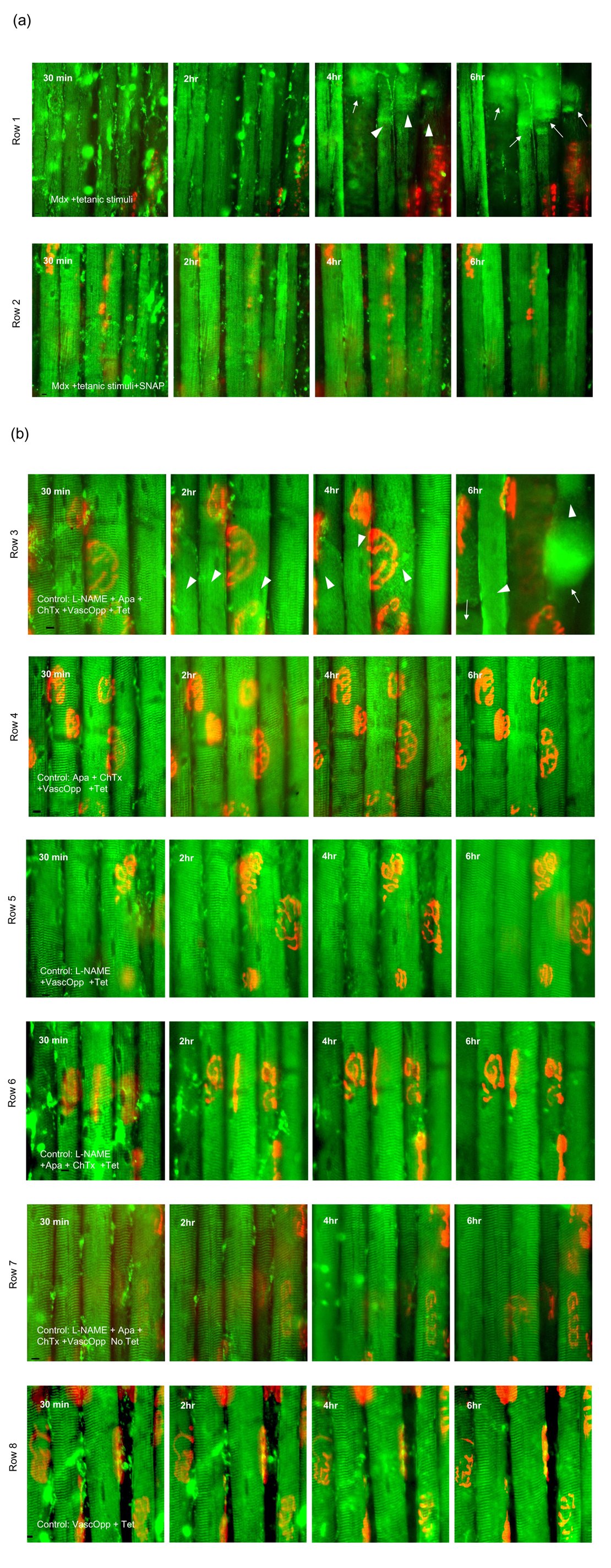

Supplement: Figure S5 — Microscopic images of myofibers from mdx mice after tetanic stimulation with our without SNAP treatment and from control mice receiving tetanic stimulation and varying degree of ischemic stress. Fluorescence images of chronological changes in the morphology of intact and dying cells in the mdx mice are shown (400x, black scale bars at the bottom of images represent 10 mm). The NMJs were stained with red fluorescence as signposts to detect individual fibers. Mitochondria and endoplasmic reticulum (M/ER) of sternomastoid muscles were stained by DiOC6. Clusters of these stained M/ER form a transverse striated pattern in the normal intact myofibers (green staining at 30 minutes). (a) In mdx mice, repeated tetanic stimuli (5 second stimulus at 50Hz, repeated 6 times with a 30 seconds interval) caused dying myofibers with an abnormal distribution of the DiOC6-stained components (a, Row1, Mdx+tetanic stimuli). At 4 hours, the M/ER showed a granular pattern of distribution in the damaged fiber loci (arrow heads). At 6 hours, those granular staining areas changed into a larger cluster of bright fluorescent spots to form a bulged structure (arrows). Administration of SNAP (NO donor, 100 mM) prevented the mdx mice fibers from undergoing contraction-induced damage (a, Row2, Mdx+tetanic stimuli+SNAP). (b) When L-NAME, apamin plus charybdotoxin, vascular oppression were all combined, tetanic stimulation caused myofiber damage in control mice (b, Row3). At 2 to 4 hours after stimulation, damaged myofibers exhibited a granular distribution pattern (arrow heads). Eventually, those damaged fibers showed bulged staining (arrows). When any of the following treatment was absent, myofiber damage did not occur to the similar extent seen in the group described above: L-NAME (b, Row4), apamin plus charybdotoxin (Row5), vascular oppression (b, Row6), tetanic stimulation (b, Row7). Tetanic stimulation and vascular oppression alone did not cause damage (b, Row8). Normal pretzel-like NMJ morpho [file pone.0000806.s005.tif]

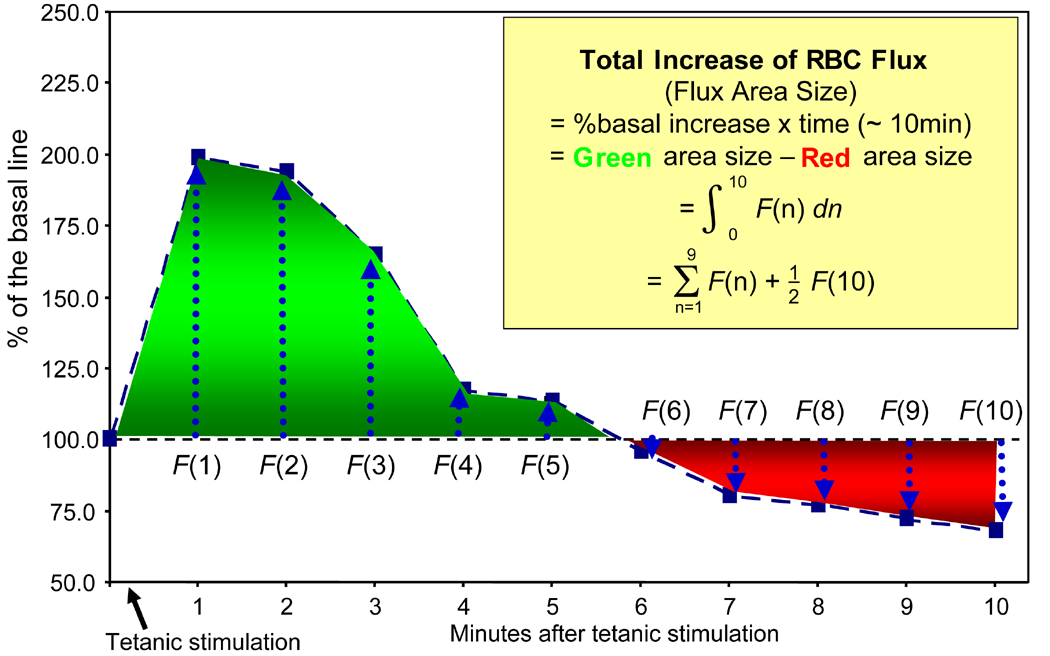

Supplement: Figure S6 — Calculation of the total increase of RBC flux. Total increase of RBC flux is given by the integral of the curve from 0 to 10 minutes after tetanic stimulation for the percent from basal RBC flux. The formula is provided as F(n)+1/2F(10): from n = 1 to 9, where F(n) stands for RBC flux (%) minus 100 at time n (minute). Note that a complete embolization of arteries will theoretically result in the value of negative 950. (2.37 MB TIF) [file pone.0000806.s006.tif]

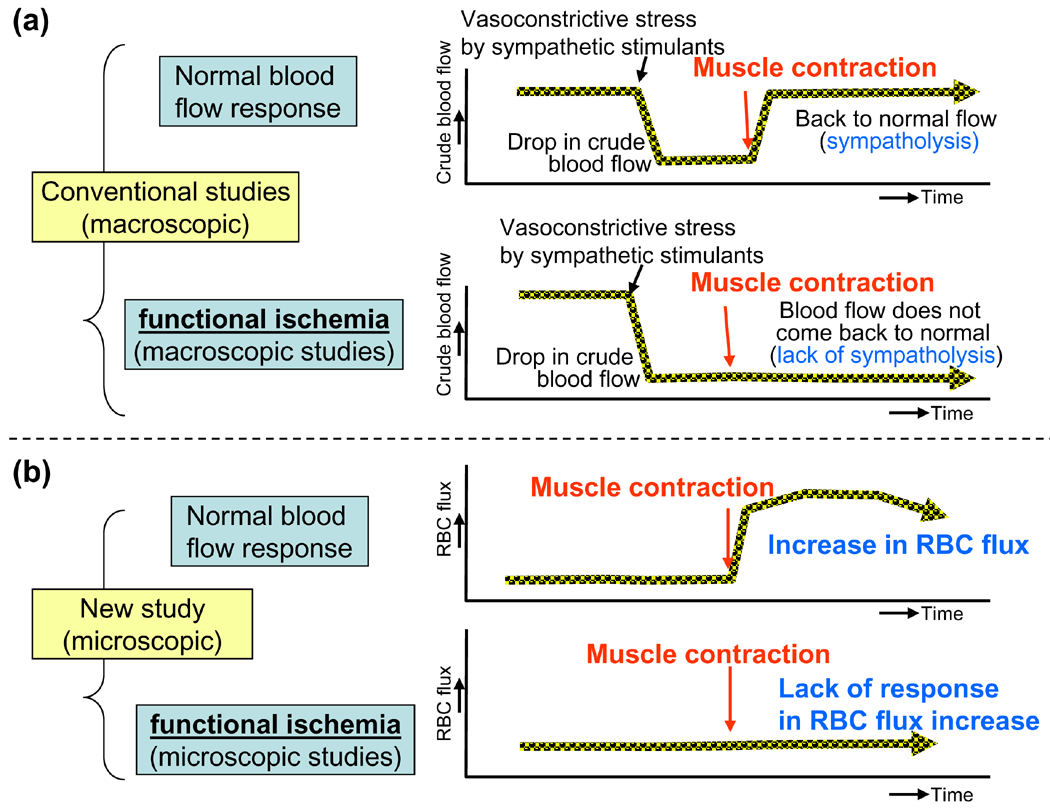

Supplement: Figure S7 — Illustration of functional ischemia by a conventional (macroscopic) and by a new (microscopic) approach. Contracting skeletal muscles require an increased blood flow to meet metabolic demands. Functional ischemia is a pathological state where this normal response is disturbed. (a) In order to observe functional ischemia, the previous studies measuring crude macroscopic blood flow required the muscles to be put under vasoconstrictive stress by sympathetic stimulants. Skeletal muscle contraction reverts the drop in the crude blood flow in normal muscles (a normal response of sympatholysis). Sympatholysis is absent in the affected muscles where normal vascular regulation mechanisms are lacking. Thus, normal muscles are rescued from ischemia by sympathetic vasoconstriction when superimposed by contractile stress but diseased muscles are put under the risk of continued ischemia. (b) In our new microscopic study, preloading with vasoconstrictive stress is unnecessary to observe functional ischemia. RBC flux increases after muscle contraction in normal subjects. This increase in RBC flux is perturbed in mdx mice. One of the technical advantages in analyzing RBC flux is to reveal a functional ischemia by a simpler scheme of stimulation than previous macroscopic studies. This observation is reasonable considering our understanding that the crude macroscopic blood flow measurement does not necessarily reflect peripheral (microscopic) RBC flux [61]. Note that RBC flux, RBC velocity, and vascular diameter are different parameters of microcirculation and may follow different kinetics, though all of these are important factors determining the state of local blood flow. We emphasize the importance of measuring the RBC flux in this type of analysis. (2.57 MB TIF) [file pone.0000806.s007.tif]

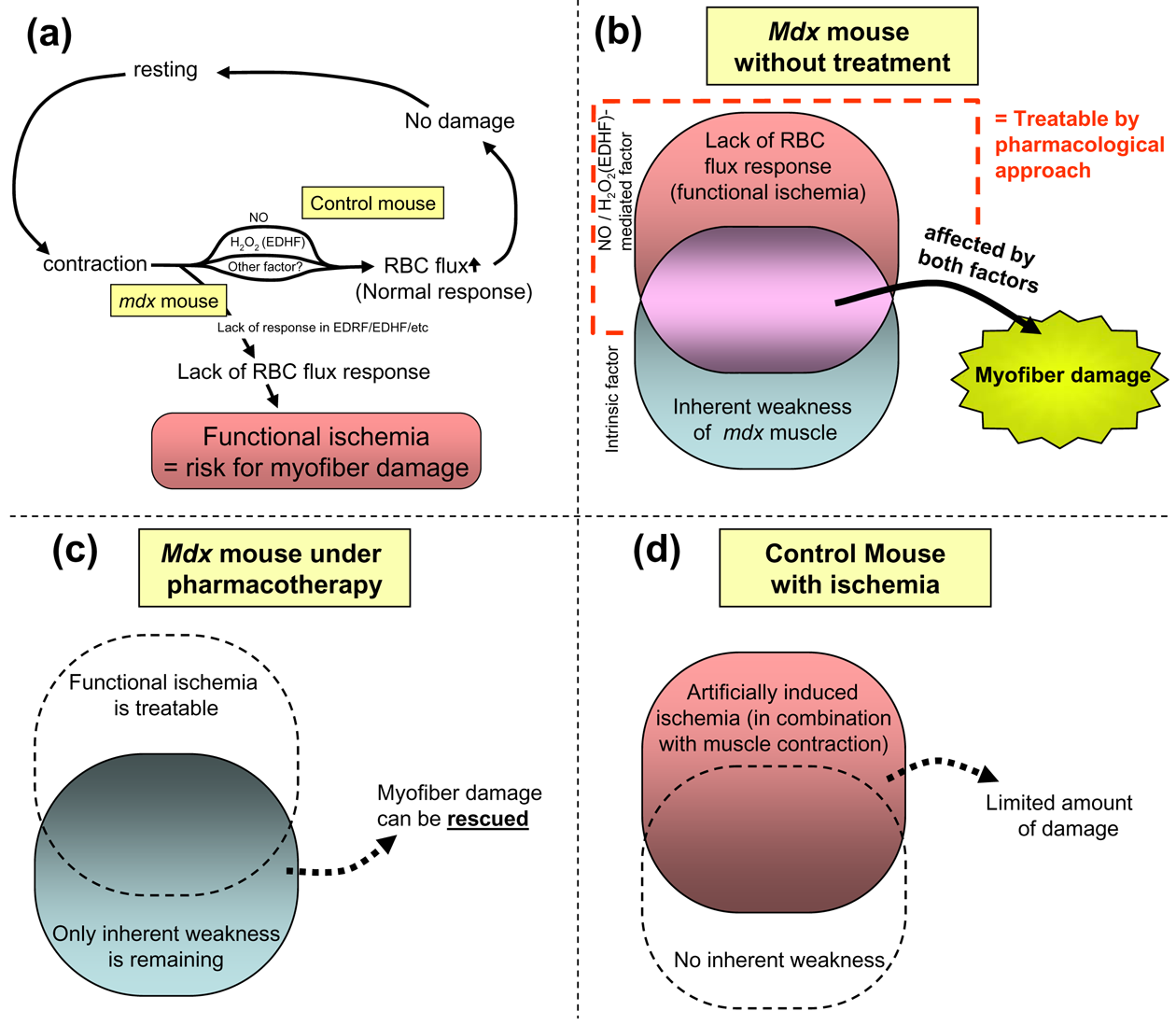

Supplement: Figure S8 — The relationship between functional ischemia and myofiber damage in control and mdx mouse models. (a) Normal blood flow response vs. lack of response in mdx mice. The results from our experiments in control and mdx mice supports the hypothesis that RBC flux in control mouse increases in response to muscle contraction via increased production of NO and H2O2, and mdx mice are deficient of NO and H2O2 production in response to muscle contraction resulting in functional ischemia and the risk for myofiber damage. (b) Quantitative analysis between the extent of functional ischemia and myofiber damage suggests that there are at least two major factors causing the contraction-induced myofiber damage in mdx mice: (1) functional ischemia, which is likely to be caused by the lack of NO/H2O2 production, and (2) a putative intrinsic factor which makes mdx myofibers inherently more vulnerable than those in control mice, independent of NO/H2O2 regulation. (c) Mdx mice under pharmacotherapy against functional ischemia are still inflicted by inherent weakness, but myofiber damage can be prevented. (d) Control mice inflicted with artificial ischemia do not show comparable amount of myofiber damage, because their myofibers are not inherently weak. (4.16 MB TIF) [file pone.0000806.s008.tif]
